# Vocal Parameters of Speech and Singing Covary and Are Related to Vocal Attractiveness, Body Measures, and Sociosexuality: A Cross-Cultural Study

**DOI:** 10.3389/fpsyg.2019.02029

**Published:** 2019-10-22

**Authors:** Jaroslava Varella Valentova, Petr Tureček, Marco Antonio Corrêa Varella, Pavel Šebesta, Francisco Dyonisio C. Mendes, Kamila Janaina Pereira, Lydie Kubicová, Petra Stolařová, Jan Havlíček

**Affiliations:** ^1^Department of Experimental Psychology, Institute of Psychology, University of São Paulo, São Paulo, Brazil; ^2^Faculty of Science, Charles University, Prague, Czechia; ^3^Faculty of Humanities, Charles University, Prague, Czechia; ^4^Department of Basic Psychological Processes, University of Brasília, Brasília, Brazil

**Keywords:** human voice, song, vocal attractiveness, fundamental frequency, sociosexuality, fitness indicators, music, voice modulation

## Abstract

Perceived vocal attractiveness and measured sex-dimorphic vocal parameters are both associated with underlying individual qualities. Research tends to focus on speech but singing is another highly evolved communication system that has distinct and universal features with analogs in other species, and it is relevant in mating. Both speaking and singing voice provides relevant information about its producer. We tested whether speech and singing function as “backup signals” that indicate similar underlying qualities. Using a sample of 81 men and 86 women from Brazil and the Czech Republic, we investigated vocal attractiveness rated from speech and singing and its association with fundamental frequency (F0), apparent vocal tract length (VTL), body characteristics, and sociosexuality. F0, VTL, and rated attractiveness of singing and speaking voice strongly correlated within the same individual. Lower-pitched speech in men, higher-pitched speech and singing in women, individuals who like to sing more, and singing of individuals with a higher pitch modulation were perceived as more attractive. In men, physical size positively predicted speech and singing attractiveness. Male speech but not singing attractiveness was associated with higher sociosexuality. Lower-pitched male speech was related to higher sociosexuality, while lower-pitched male singing was linked to lower sociosexuality. Similarly, shorter speech VTL and longer singing VTL predicted higher sociosexuality in women. Different vocal displays function as “backup signals” cueing to attractiveness and body size, but their relation to sexual strategies in men and women differs. Both singing and speech may indicate evolutionarily relevant individual qualities shaped by sexual selection.

## Introduction

Speech and singing are among the most common vocal productions in adult humans and their presence seems to be universally shared across modern human populations (Brown, 1991). It is assumed that they have a common ancestor (Brown, 2001, 2017; Mithen, 2005) which evolved into two specialized systems of structured vocal communication (Lehmann et al., 2009). It also seems that prosody, the musical part of speech which conveys mainly emotional information, is rooted already in the origins of both spoken and sung vocal production (Filippi, 2016; Brown, 2017). It has recently been shown that speech and singing may have diverged from a protolanguage and split in two systems based on their communicative function. In particular, when referential and emotional functions are introduced into an artificial communication system, the system diverges into speech- and music-like vocalizations, respectively (Ma et al., 2019). Moreover, despite a vast variability across cultures, the function of specific kinds of songs (e.g., a love song) is cross-culturally comprehensible based on their structural form (Mehr et al., 2018). Interestingly, both human and bird songs tend to employ similar descending/arched melodic contour despite substantial differences in absolute pitch and duration, which indicates similar underlying motor constraints across cultures and species (Savage et al., 2017).

Singing and speech differ in the use of vocal anatomy (Sundberg, 1977, 2018), require different patterns of breathing (Leanderson et al., 1987), and neuroanatomy of production and appreciation is likewise specific to each of the two domains (Zatorre and Baum, 2012). Cognitive processing of speech and singing is also specific for each domain, as shown in patients with amusia who have intact speech processing and patients with aphasia who have no impairment of musical capacities (Peretz and Coltheart, 2003). Despite the different design features, such as the arbitrariness of speaking and regular beat and discrete set of pitches in singing, the two domains share some further features, such as hierarchical structure and complexity (Fitch, 2006). Moreover, both speaking and singing voice provide relevant information about the producer’s gender, identity, location, emotional state, and behavioral tendencies (Weninger et al., 2011) and individuals can identify others based on their speech and singing (Trehub et al., 2009).

While spoken language is mostly specific to humans and language-like forms of vocalization exist in a few other animals (prairie dogs, dolphins, etc.) (Slobodchikoff et al., 1991; Janik, 2013), singing has its parallels in many other species. The capacity for learning complex songs, new sequences and sounds has arisen independently in birds (songbirds, hummingbirds, and parrots) and mammals (whales, seals, and humans) (Fitch, 2005). Since Darwin’s (1871) groundbreaking works, sexual selection has been viewed as one of the most important factors that drove the evolution of singing as a way of attracting the opposite sex and advertising individual qualities. There is a large body of research showing the importance of singing in mating success across various avian and mammalian species (e.g., Searcy and Andersson, 1986). In some species, singing seems to function as an honest signal of underlying individual qualities, so that e.g., lower-pitched songs advertise a larger body size (Hall et al., 2013). In humans, irrespective of their original adaptive value, speaking and singing can likewise be considered honest signals that meet the four requisite criteria (Smith and Bird, 2000). They both require a long time for maturation, practice, and learning (Welch, 2006), their production is energetically costly because they rapidly fade (Fitch, 2006), they can suffer from noise interference, and require intense breathing (Leanderson et al., 1987). Both speech and singing are easily perceptible by most people, are used in mating-relevant contexts, such as courtship (White et al., 2018), can increase individual mating success, and both can serve as cues to genetic qualities of the producer (Miller, 2000). There are also some significant differences between the two: singing requires higher vocal control (Zarate, 2013) and is more demanding than speech because singers need to tailor the subglottal pressure to both pitch and loudness (Sundberg, 1977, 2003). Singing can also be louder than speech, involving more muscle activity (Åkerlund and Gramming, 1994; Leanderson et al., 1987), and it includes a performative context (Fitch, 2006) which attracts more attention and is thus socially riskier. People even tend to abbreviate their singing performance in front of supposedly expert audience (Garland and Brown, 1972). It is thus well possible that singing is even harder to fake as an honest signal of underlying individual qualities than speech is, thus serving as an ornament that can affect the quantity or quality of sexual partners.

Human voice plays an important role in mate preferences and intrasexual competition (Puts, 2010; Pisanski and Feinberg, 2019), but so far, most research on human voice attractiveness and its indicators focused on speech. Some vocal parameters, especially the fundamental frequency (F0), differ between males and females of many species, with humans exhibiting an even greater sexual dimorphism than other primates (Puts et al., 2016). F0 is produced by vibrations of the vocal folds within the larynx and together with the corresponding harmonics is perceived as voice pitch (Pisanski et al., 2016). On average, men produce lower-pitched voices than women: this is due to the effects of testosterone during puberty which thickens and lengthens male vocal folds and thereby lowers the F0 (Pisanski and Feinberg, 2019). From a more general perspective, vocal sexual dimorphism is supposed to be at least in part the result of intrasexual competition, especially in the context of male-male competition (e.g., Puts, 2010). Indeed, men with lower-pitched voices are perceived as older, taller, heavier, more masculine, and more dominant than men with higher-pitched voices (Collins, 2000; Feinberg et al., 2005; Puts et al., 2006, 2007; Pereira et al., 2019). And similarly, women with lower-pitched voices are perceived as more dominant (Borkowska and Pawlowski, 2011), and both men and women with lower-pitched voices reported higher leadership capacities (Klofstad et al., 2012).

Aside from intrasexual competition, intersexual selection may have also played a role in shaping sex differences in voice. There is robust evidence that women prefer relatively low-pitched male speaking voices, while men prefer relatively high-pitched female voices (for a review, see Pisanski and Feinberg, 2019). Nevertheless, the relationship between male and female F0 and attractiveness is non-linear: the most attractive male voices are around 96 Hz and the most attractive female ones up to 280 Hz (Borkowska and Pawlowski, 2011; Saxton et al., 2015). Importantly, preferences for lower- and higher-pitched voices in men and women, respectively can be specific to certain contexts and individuals, such as short-term relationships (Little et al., 2002), coupled women (Valentová et al., 2013), and nulliparous women (Apicella and Feinberg, 2009), and in some populations that can even be inverted (Shirazi et al., 2018). Moreover, recent evidence suggests that lower-pitched female voices are perceived as attractive (Babel et al., 2014), and women actively lower their voices when speaking to attractive men or when willing to sound attractive (Hughes et al., 2014; Pisanski et al., 2018; but see Fraccaro et al., 2011). Lower pitched voices in women can thus signal their immediate interest and/or sexual appetence.

In line with the fitness indicator hypothesis within the sexual selection theory, vocal characteristics can convey information about the underlying qualities of voice producers, e.g., information about their health and reproductive potential. For example, men with relatively low-pitched voices exhibit low cortisol and high testosterone levels, which are related to immunoreactivity (Evans et al., 2008; Hodges-Simeon et al., 2015; Puts et al., 2016). Moreover, among North American men, a lower-pitched voice is associated with more female sexual partners (Puts, 2005), and lower-pitched male Hadza hunter-gatherers have on average a higher number of offspring (Apicella et al., 2007). Furthermore, both men and women with more attractive voices reported more sexual partners, extra-pair copulations, and earlier age of the first sex (Hughes et al., 2004), which are all considered proxies of potentially higher reproductive success.

Moreover, voice attractiveness is associated with several body measures that develop under the influence of sex-specific hormones and are thus viewed as indicators of genetic and developmental quality, and subsequently also the reproductive fitness of the individual. For example, voice attractiveness is positively associated with the shoulders-to-hip ratio in men and negatively associated with the waist-to-hip ratio in women (Hughes et al., 2004). Low pitched male voices are linked to larger body size, especially weight and height, to a particular body shape (shoulder and chest circumference, shoulder-to-hip ratio) (Evans et al., 2006), and arm strength (Puts et al., 2011). Nevertheless, a recent meta-analysis had shown that compared to other vocal parameters, voice pitch is not a reliable predictor of height in adults of the same sex (Pisanski et al., 2014) and it is a poor predictor of body weight, shape, and strength (Collins, 2000; Collins and Missing, 2003; Bruckert et al., 2006; Evans et al., 2006; Sell et al., 2010; Vukovic et al., 2010; Pisanski et al., 2016; Raine et al., 2019).

Formants, on the other hand, which are the resonant frequencies of the vocal tract, are more constrained by the anatomical structures related to body size. Formants are anatomically and functionally dissociated from fundamental frequency and are therefore a more reliable indicator of body size and shape both in humans and in numerous other mammalian species (Pisanski et al., 2014). Formants are also sexually dimorphic, whereby men show lower formant frequencies than women (Pisanski et al., 2016). Individuals who produce lower formant frequencies are perceived as more physically dominant (Puts et al., 2007) and women who produce higher formant dispersion are perceived as flirtatious and attractive by both men and women (Puts et al., 2011). Individual vocal characteristics thus may provide cues to different bodily traits and sexual behaviors linked to individual’s potential reproductive success.

Importantly, voice is a dynamic behavioral display which can be both intentionally and involuntarily modulated under specific situations so as to express or exaggerate ecologically relevant traits, including emotions (Pisanski et al., 2016). For example, both men and women change their voice when speaking to infants (Foulkes et al., 2005; Broesch and Bryant, 2015) and this specific infant-directed speech affects attention and communicative outcomes of the children (Rowe, 2012; Spinelli et al., 2017). Similarly, women modulate voice pitch when speaking to attractive men (Fraccaro et al., 2011; Hughes et al., 2014; Pisanski et al., 2018) and voices of both men and women who speak to an attractive individual are perceived as more attractive by others (Leongómez et al., 2014). Also, people can volitionally increase their vocal tract length (as estimated from formant frequencies) and decrease fundamental frequency to imitate a larger body size, and vice versa (Pisanski et al., 2016). The overall prosody of speech can be effectively modulated when expressing different emotions, such as high, loud, and fast prosody while feeling happy, and the opposite pattern while being sad (for review, see Brown, 2017). Interestingly, the same vocal modulation appears when expressing emotions by music, which suggests that both displays may convey similar information (Juslin and Laukka, 2003; Zatorre and Baum, 2012).

Although both singing production and perception is a scientific research field in its own right (Sundberg, 2003), singing accuracy is related to several loci on chromosome 4 and exhibits 40% heritability (Park et al., 2012), and singing frequently features in mating contexts (e.g., as serenades and love songs, see Dukes et al., 2003; Levitin, 2008), it tends to be overlooked by psychological research on voice attractiveness. As an exception, one study found that women who were judged as good singers based solely on the audio recordings were also independently rated as more attractive based on soundless video recordings (Wapnick et al., 1997). This is in line with research which shows that in women, attractiveness and masculinity-femininity ratings based on different modalities are correlated (e.g., Valentova et al., 2017c; Pereira et al., 2019). Nevertheless, further research is needed to test to what extent are the perceptual characteristics of speech and singing voice intercorrelated and whether both vocal displays function as backup signals, i.e., as signals that indicate similar underlying qualities, rather than multiple messages, i.e., signals that indicate different qualities of individuals (see Johnstone, 1996; Bro-Jørgensen, 2010). To the best of our knowledge, only one study tested the attractiveness of speech and singing in women and it concluded that attractiveness rated from both vocal displays is correlated and in both cases increases with voice pitch (Isenstein, 2016). This can be viewed as indicating that different vocal displays may serve as backup signals.

### Aims of the Current Study

In the current study, we tested whether certain perceptual singing and speaking characteristics (perceived attractiveness, voice pitch, and formant frequencies) serve as cues to specific individual physical and behavioral qualities. Since singing production is more costly than speech, one could predict that the perceived attractiveness of singing would be a stronger indicator of individual quality than the attractiveness of speech. We have therefore tested the association between the attractiveness of singing and speech and selected body fitness indicators (body size and shape). We have also tested the relation between attractiveness ratings of both vocal displays and sociosexuality, which we used as a proxy of a short-term sexual strategy that may, especially in men, lead to increased reproductive success. We have further investigated how selected vocal parameters (voice pitch and vocal tract length as estimated from formant frequencies) mediate the possible associations between the vocal attractiveness, body cues, and sociosexuality.

Further, we tested whether the capacity to modulate the voice and singing experience may influence the rated vocal attractiveness. We hypothesized that both singing experience and a higher ability to modulate voice would lead to a more attractive vocal production.

Additionally, we tested for possible differences in vocal parameters between the sexes in two distinct populations, a Brazilian and a Czech one. So far, very little cross-cultural research has been conducted on evolutionarily relevant aspects of voice characteristics and perceptions. Majority of that research was conducted in the United States, Western and Central Europe (for review, see Pisanski and Feinberg, 2019). Studies comparing more populations with different physical, cultural, and linguistic compositions are thus needed to increase generalization of results. For example, although most North American and European studies concluded that women prefer lower-pitched male voices, Filipino women seem to follow the opposite pattern (Shirazi et al., 2018). In our study, we employed two sets of participants using sampling in one South American and one Central European population (Brazil and Czech, respectively), which differ widely as to their history, culture, ethnicity, and demographic data, and which both also differ from Western European and North American societies. Moreover, these populations also differ in several body measures, such as height and weight (e.g., Varella et al., 2014; Valentova et al., 2016), facial and body hair in men (Valentova et al., 2017b), while self-rated breast size, buttock size, and WHR in women is the same in both (Valentova et al., 2017a). Furthermore, Brazilian population reports a significantly higher sociosexuality than the Czech population (Varella et al., 2014). Both populations are also linguistically different: Brazilian Portuguese is a Latin language while Czech belongs to Slavic languages. Previous studies reported that several vocal parameters differ between the different linguistic groups (Mennen et al., 2012). The two populations thus offer an interesting opportunity to analyze vocal production and perception and its relation to body measures and sociosexuality.

## Methods

### Target Participants

The final sample was composed of 40 Brazilian men (M = 23.70 years; SD = 3.67, range 19–34) and 44 women (M = 23.91 years; SD = 4.99, range 18–35) recruited at the University of São Paulo, in São Paulo city, and 33 Czech men (M = 22.45 years; SD = 2.35, range 18–28) and 35 women (M = 22.37 years; SD = 2.57, range 19–29), recruited at the Charles University, Prague. We selected predominantly heterosexual participants (0–2 on a Kinsey scale) because individuals with different sexual orientations can show variation in several vocal parameters (Kachel et al., 2018) which can be detected even by naïve listeners (Valentova and Havlíček, 2013).

### Procedure

In both countries, each participant consented to take part in a broader study (see, Varella et al., 2014; Valentova et al., 2017c). Participants completed questionnaires, we took body measurements, standardized facial and body photographs, and recorded videos of both speech and singing. Only data relevant for this study are described below. Brazilians are not allowed to receive financial reward but Czech participants received remuneration amounting to 300 CZK (approximately 13 USD). The project was approved by the Charles University IRB (2011/07).

### Questionnaires

Participants completed a sociodemographic questionnaire and the Revised Sociosexual Orientation Inventory (SOI-R; Penke and Asendorpf, 2008). The SOI-R measures an individual’s willingness to engage in uncommitted sex. It consists of nine items (e.g., “With how many different partners did you have sexual intercourse on one and only one occasion?”), loading into three subscales of sociosexual behavior, attitudes, and desire. They also answered, on a 10-point scale, how much they liked to sing (1 = not at all, 10 = very much). We used this information as a motivational factor that may influence singing frequency, singing training, and thus singing experience, as shown in Busch (2013).

### Vocal Recordings

Vocal samples were recorded under standardized conditions, in a closed and quiet room, and all by one researcher. For all recordings, we used a professional digital stereo Olympus LS-100 Multi-Track Linear PCM recorder, whereby the participants’ lips were approximately 10 cm from the microphone. When performing the vocal tasks, all participants were seated on a chair. First, participants were informed about the whole recording procedure: this information was printed for them. After a small vocal exercise to warm-up the voice and get used to being recorded, participants read a short sentence using standardized names across all participants. In Brazil, all men and women, respectively, pronounced “*Oi, meu nome é Pedro/Ana, e eu sou de Belo Horizonte*,” while Czech men and women, respectively, said “*Jmenuji se Petr/Petra a pocházím z Havlíčkova Brodu*” (*Hi, my name is Petr/Pedro/Petra/Ana and I come from Belo Horizonte/Havlíčkův Brod*). Subsequently, they sang the first part of “Happy Birthday” (in the Brazilian Portuguese version “*Parabéns para você, nesta data querida, muitas felicidades, muitos anos de vida*,” in the Czech version “*Hodně štěstí zdraví, hodně štěstí zdraví, hodně štěstí, milý Honzo, hodně štěstí zdraví*”). Finally, they first read and then sang the first stanza of their national anthem (the verbal content of speech and singing was thus matched).

To minimize raters’ overload, we extracted parts of the national anthem using SoundForge 8.0 software. In the Brazilian sample, we extracted the first two lines of the national anthem (“*Ouviram do Ipiranga as margens plácidas, de um povo heróico o brado retumbante*”), while for the Czech participants, we extracted the third and fourth line, which unlike the first two lines are not repetition of each other (“*Voda hučí po lučinách, bory šumí po skalinách*”). Only these recordings were subsequently rated by independent participants and analyzed for vocal parameters. All participants spoke their native language, i.e., either Brazilian Portuguese or Czech. None of the participants reported any serious vocal or respiratory problem at the time of the data collection.

Happy Birthday was selected because it is cross culturally known and commonly sung in intimate and emotionally loaded social situations, usually with the family, friends, and romantic partners, and it has been used in research on singing previously (e.g., Christiner and Reiterer, 2013). The national anthem is also widely known within each country, it is relatively unconnected to mating context and is thus more neutral.

Recordings were analyzed using Praat software (Boersma and Weenink, 2013) for mean, minimal, and maximal fundamental frequency (F0), and the first four formants (F1–F4). F0 is the rate of vocal folds vibration perceived as an overall voice pitch. We used an autocorrelation algorithm with parameters set to a pitch floor of 75 Hz and pitch ceiling of 300 Hz for men, and a pitch floor of 100 Hz and pitch ceiling of 500 Hz for women, because these are the appropriate boundaries for analyzing adult voices recommended by the software developers (Boersma and Weenink, 2013). All other values were set to default. Average speech F0 per recording ranged between 92.47 (Corresponding to musical note F#_2_, here F note is heightened by semitone, which is indicated by #) and 177.70 Hz (F_3_) in men, and between 164.10 (E_3_) and 253.10 Hz (B_3_) in women. For singing, F0 ranged between 103.60 (G#_2_) and 208.50 Hz (G#_3_) in men, and between 168.5 (E_3_) and 348.20 Hz (F_4_) in women. All F0 were transformed to perceptual pitch expressed in a semitone difference between A4 (440 Hz) and F0 using a standard formula 12*log*_2_ (F0/440). This scale is based on standard music notation and reflects the logarithmic nature of human pitch perception, where both A_3_ (−12, 220 Hz) and A_5_ (12, 880 Hz) are at an equal octave distance (12 semitones) from A_4_. We subtracted the minimal F0 from the maximal F0 of each recording to obtain its perceptual range in semitones. Average speech range per recording ranged between 4.61 and 21.07 semitones in men and between 5.34 and 27.61 semitones in women, while the singing range ranged between 6.76 and 23.74 semitones in men, and between 8.76 and 27.84 semitones in women. F0 and ranges were averaged for each participant across recordings for speech and singing separately.

Apparent vocal tract length (VLT) was calculated from the first four formants (F1–F4) according to a formula described in Pisanski et al. (2014). F1 to F4 were measured in Praat using semiautomated approach. First, recordings were preprocessed by Vocal Toolkit’s “Extract voiced and unvoiced” script (Corretge, 2019) and subsequently only the voiced parts were used for further formants analysis. Second, formants were analyzed by Burg method with recommended preset values and maximum formant levels of 5000 and 5500 Hz for men and women, respectively. In each recording from the list of results were omitted readings suggesting presence of silence and erroneous readings. F1 to F4 levels are represented by median of remaining formants readings.

Subsequently, formant spacing (Δ*F*) was estimated as a slope of the linear regression line with an intercept set to 0 from a relationship

$$F_{i}=\frac{\left(2\,i-1\right)}{2}\,\Delta \,F$$

where “*i”* refers to the formant number. Apparent vocal tract length was derived from formant spacing using

$$V\,T\,L\,\left(\Delta \,F\right)=\frac{c}{2\,\Delta \,F}$$

where *c* = 33.500 cm/s is the speed of sound in a uniform tube with one end closed.

### Anthropometry

We measured participants’ body height in centimeters, weight in kilograms, and body characteristics previously found to be associated with vocal attractiveness, namely the circumference of the shoulders, waist, and hips (Dixson et al., 2003; Stulp et al., 2013; Valentova et al., 2014, 2016, 2017a). Then we computed the waist-to-shoulder ratio (WSR) in men and waist-to-hip ratio (WHR) in women (for details on the procedure, see Varella et al., 2014).

### Vocal Ratings

An independent sample of heterosexual raters anonymously judged voice attractiveness of all vocal recordings of individuals of the opposite sex on a 7-point scale (1 = not at all attractive, 7 = very attractive) using Rater software (facelab.org). All raters reported being predominantly heterosexual (0–2 on a Kinsey scale). Brazilian raters (51 men: M = 22 years, SD = 3.4 years; 59 women: M = 22.1 years, SD = 3.4) were recruited among the students of the University of Brasília, while the Czech raters (46 men: M = 21.7 years, SD = 1.9; 47 women: M = 20.6 years, SD = 1.1) were recruited at the Charles University, Prague. The rating took place in an empty classroom, each voice recording containing the relevant phrase was presented only once using headphones and with unmanipulated volume. Each rater evaluated either all Brazilian or all Czech recordings. For instance, one Brazilian rater rated all Czech recordings, while another Brazilian rater rated all Brazilian recordings. The recordings were divided into eight blocks (two speech and two singing recordings, Brazilian and Czech sample) and randomized within each block. Interrater agreement (Cronbach’s α) was high in all recording × rater set combinations (min α = 0.79) (For a full overview of Cronbach’s α, see Supplementary Material). Pearson correlations between average attractiveness ratings of Czech and Brazilian raters were high for both speech [*r* = 0.694, 95%CI (0.602,0.768) *p* < 0.001] and singing [*r* = 0.788, 95%CI (0.719,0.841) *p* < 0.001]. We have therefore used as a unit of analysis the mean rating of attractiveness for each target across all raters.

### Statistical Analyses

All analyses were conducted using R 3.5.1 software, and SPSS version 21 (IBM Corp., Armonk, NY, United States). To explore associations between the measured and rated voice parameters in speech and song, we ran parametric correlations (Pearson correlation) and paired *t*-tests to test for possible differences between the two vocal displays.

Relationships between the four exogenous variables (waist-to-hip or waist-to-shoulders ratio, height, weight, and age), mediating acoustic qualities (speech and singing F0 and range), speech and singing attractiveness, and the total sociosexuality score were investigated using path analysis. The structural model contained 6 correlations and 38 regression coefficients. Analysis was conducted using sem() function from the lavaan package. Because of small parameters/observations ratio (as low as 1.66 in the male sample), robust *p* values were obtained using Monte Carlo simulation. The distribution of expected correlation/regression coefficients was derived from 10,000 simulation runs, where the full model was estimated on a randomized dataset. The issue of influential points was avoided by jackknife resampling. Removing one observation at a time, we extracted sets of all measures including standardized model estimates and *p* values. Coefficients which remained significant regardless of the removed data points are emphasized in the main article, while full results are reported in the Supplementary Material. Path invariance was tested from the χ^2^ difference between configural invariant, where structure is restricted to be equal between the groups, and path invariant, where all coefficients are restricted to be equal between the groups, with degrees of freedom corresponding to the number of estimated parameters. Path invariance was evaluated between men and women and subsequently between Czech and Brazilian participants within each sex. Interrater agreement was evaluated using Cronbach’s α calculated using alpha() function from the psych package (the code is available at https://github.com/costlysignalling/Speech-and-singing-attractiveness).

Further, to test for the possible effect of voice experience on rated voice attractiveness, we assessed non-parametric correlations (Kendall rank correlation indicated by coefficient τ) between the rated attractiveness of both spoken and sung recordings and how much the participants liked to sing. To test the voice modulation hypothesis, we computed the absolute difference between singing and speaking F0, singing and speaking F0 range, and the absolute difference between singing and speaking VTL, which gave us an index of (dis)similarity of these vocal parameters between the two vocal displays. The higher the absolute difference, the larger the difference between speech and singing, and thus the higher vocal modulation. We further correlated these absolute differences with attractiveness ratings, separately for men and women. In these analyses, we did not control for multiple comparisons across tests, because the samples were independent.

Additionally, we used General Linear Models (GLM) to test for possible effects of sex, age, and country on voice attractiveness ratings. Similarly, to test whether mean F0, range F0, and VTL of speech and singing differ between men and women or between Brazilian and Czech participants, we performed a multivariate GLM with mean F0 and F0 range as dependent variables and sex and country of targets as factors. Due to a limited samples size, we evaluated only simple models. The effect size displayed is a partial Eta-squared (η_p_^2^).

## Results

### The Effect of Targets’ Sex and Country on Spoken and Sang F0, F0 Range, and VTL

We found large effects of targets’ sex on all vocal parameters; mean speech F0 (F = 1074.30, df = 1, 153, *p* < 0.001, η_p_^2^ = 0.878), mean speech F0 range (F = 14.12, df = 1, 153, *p* < 0.001, η_p_^2^ = 0.086), VTL as measured from speech (F = 2114.02, df = 1,153, *p* < 0.001, η_p_^2^ = 0.934), mean singing F0 (F = 736.84, df = 1, 153, *p* < 0.001, η_p_^2^ = 0.831), mean singing F0 range (F = 7.00, df = 1, 153, *p* = 0.009, η_p_^2^ = 0.045), and VTL as measured from singing (F = 1537.91, df = 1, 153, *p* < 0.001, η_p_^2^ = 0.911). Estimated marginal means revealed that women had a higher F0 and F0 range and shorter VTL than men (for mean values, see Table 1). There was also a significant effect of the target country on speech F0 range (F = 4.31, df = 1, 153, *p* = 0.040, η_p_^2^ = 0.028), VTL as measured from speech (F = 10.49, df = 1,153, *p* = 0.001, η_p_^2^ = 0.065), and VTL as measured from singing (F = 6.59, df = 1, 153, *p* = 0.011, η_p_^2^ = 0.042). Estimated marginal means show that Czech participants had a lower speech F0 range and longer VTL than the Brazilian participants (see Table 1 for details).

**TABLE 1:** Mean fundamental frequency (F0) and the range of fundamental frequency (F0 range) in semitones, and VTL (in centimeters) in men and women.

	**Men**			**Women**		
	**Brazilian**	**Czech**	**Total**	**Brazilian**	**Czech**	**Total**
	**(*N* = 42)**	**(*N* = 35)**	**(*N* = 77)**	**(*N* = 45)**	**(*N* = 36)**	**(*N* = 81)**
Mean F0 – speech (SD)	−22.15(2.13)	−22.63(1.84)	−22.37(2.01)	−13.19(1.28)	−13.00(1.53)	−13.11(1.39)
Mean F0 – singing (SD)	−19.71(2.49)	−20.51(2.26)	−20.07(2.40)	−10.50(2.05)	−10.07(2.08)	−10.31(2.06)
Mean F0 range – speech (SD)	11.98 (2.52)	11.02 (2.90)	11.55 (2.72)	14.25 (4.23)	12.97 (3.55)	13.69 (3.97)
Mean F0 range – singing (SD)	14.65 (2.52)	14.24 (2.42)	14.47 (2.46)	15.70 (3.25)	15.74 (2.96)	15.72 (3.10)
VTL – speech (SD)	17.32 (0.49)	17.57 (0.51)	17.44 (0.51)	14.18 (0.30)	14.38 (0.39)	14.27 (0.35)
VTL – singing (SD)	16.80 (0.53)	17.18 (0.64)	16.98 (0.61)	13.93 (0.38)	13.95 (0.34)	13.94 (0.36)

It is worth noting that the average VTL measures for men and women (Table 1) compare to population-level averages (Pisanski et al., 2014).

### Comparisons Between Speaking and Singing Voice

F0 measured from speech was strongly positively correlated with F0 measured from singing in both men (*r* = 0.800, *N* = 73, *p* < 0.001) and women (*r* = 0.607, *N* = 79, *p* < 0.001). F0 range measured from speech was correlated with F0 range measured from singing in men (*r* = 0.408, *N* = 73, *p* < 0.001) but not in women (*r* = 0.160, *N* = 79, *p* < 0.159). Vocal tract length (VTL) as estimated from formant frequencies was strongly positively correlated between speech and singing in both men (*r* = 0.808, *N* = 81, *p* < 0.001) and women (*r* = 0.764, *N* = 85, *p* < 0.001). Vocal attractiveness rated from speech and singing was also strongly positively correlated in both men (*r* = 0.720, *N* = 73, *p* < 0.001) and women (*r* = 0.674, *N* = 79, *p* < 0.001). Paired *t*-test revealed that voices rated from speech were judged significantly higher on attractiveness than voices rated from singing in both men (*t* = 6.66, df = 72, *p* < 0.001) and women (*t* = 3.85, df = 78, *p* ≤ 0.001).

### Structural Models

The model which analyzes the fundamental frequency is not path-invariant with respect to the sex of individuals (χ^2^ = 117.03, df = 44, *p* < 0.001) but is path-invariant with respect to participants’ nationality (χ^2^ = 49.58, df = 44, *p* = 0.26 in men, χ^2^ = 60.68, df = 44, *p* = 0.05 in women). Results are therefore reported separately for men and women but jointly for Czech and Brazilian participants.

**FIGURE 1:**
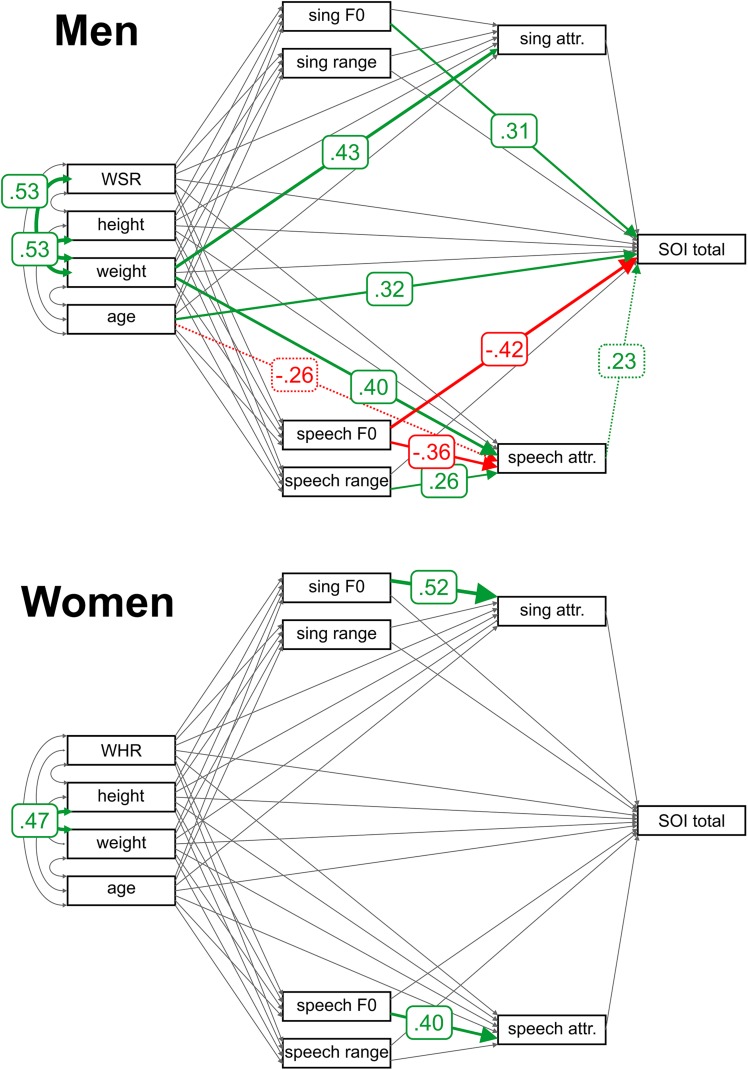
Path analysis results for F0. Arrows represent estimated parameters. Relationships significantly different from 0 (indicated by robust permutation yielded *p* values) are colored (positive relationships in green, negative in red) and labeled with standardized model estimates. Relationships that failed to meet the jackknife significance stability criteria are represented with a dashed line. F0 = average fundamental frequency; WSR = waist-to-shoulder ratio; and WHR = waist-to-hip ratio.

Using path analysis (see Supplementary Tables S6, S7 for full models), we found that in men, lower-pitched speech was rated as more attractive (Figure 1). The same held of singing, but this relationship did not reach statistical significance. In men, a broader speech range, but not singing range, was rated as more attractive. Attractive speech was positively associated with the total SOI, but this relationship failed to maintain its stability in jackknife resampling. The total SOI was directly connected to a lower F0 in speech and higher F0 in singing. Body weight had a strong and positive direct effect on perceived speech and singing attractiveness. Age had a negative effect on speech attractiveness but the effect failed to remain stable under jackknifing (see Supplementary Table S8).

Higher-pitched female voices (both in speech and singing) were rated as more attractive. No other relationship except for correlation between height and weight was significant (see Supplementary Tables S7, S9).

The additional model that analyzed vocal tract length (VTL) was not path-invariant with respect to the sex of individuals (χ^2^ = 109.44, df = 44, *p* < 0.001) but was path-invariant with respect to participants’ nationality at least in women (χ^2^ = 66.99, df = 44, *p* = 0.01 in men, χ^2^ = 59.18, df = 44, *p* = 0.06 in women). Results are reported separately for men and women but jointly for Czech and Brazilian participants for a better comparison with the original model that employs the F0.

**FIGURE 2:**
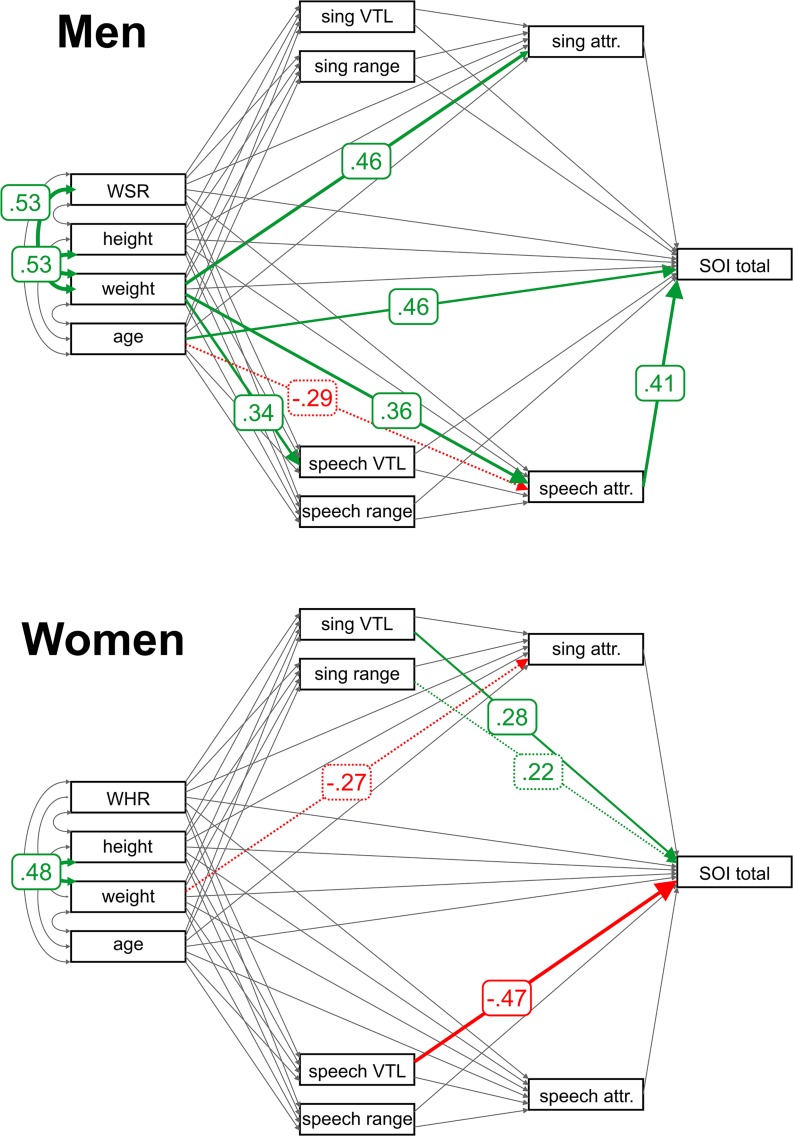
Path analysis results for VTL. Arrows represent estimated parameters. Relationships significantly different from 0 (indicated by robust permutation yielded *p* values) are colored (positive relationships in green, negative in red) and labeled with standardized model estimates. Relationships that failed to fulfill the jackknife significance stability criteria are represented with a dashed line. VTL = apparent vocal tract length; WSR = waist-to-shoulder ratio; and WHR = waist-to-hip ratio.

Many relationships in the structural model remained similar when we replaced average F0 with apparent VTL (Figure 2). Nevertheless, the VTL failed to predict speech or singing attractiveness reliably. In women, we observed a reverse relationship between speech and singing VTL and the total SOI. In this model, however, these relationships were stronger because the potentially mediating path between VTL and attractiveness was weaker. This was possibly due to the fact that in the first model, which relied on average fundamental frequency together with the F0 range, both measurements of vocal quality were based on the same characteristic (F0 – either as average or as a difference between minimum and maximum), which in effect allowed us to partition out their respective contributions to speech and singing attractiveness better. The model with VTL, which tightly correlated with average F0, lowered the partial correlations beyond the threshold of statistical significance. All the relationships were, however, in the direction that would be expected based on the strong negative correlation between VTL and mean F0 (See Supplementary Tables S10–S12).

### The Effect of Singing Experience and Voice Modulation on Voice Attractiveness

Non-parametric correlations showed a positive association between how much men liked to sing and attractiveness as rated from both speech (τ = 0.253, *N* = 87, *p* < 0.001) and singing (τ = 0.277, *N* = 87, *p* < 0.001). In women, this association was rather weak and significant only in singing attractiveness (τ = 0.171, *N* = 90, *p* = 0.024) but not in speech attractiveness (τ = 0.101, *N* = 91, *p* = 0.183). Furthermore, the absolute difference of F0 between speech and singing was positively correlated with how much men and women liked to sing (τ = 0.255, *N* = 90, *p* = 0.001; τ = 0.281, *N* = 93, *p* < 0.001, respectively). Moreover, the absolute difference of F0 was positively associated with rated singing attractiveness in both men (τ = 0.177, *N* = 87, *p* = 0.015) and women (τ = 0.294, *N* = 90, *p* < 0.001) but not significantly associated with speech attractiveness in either men (τ = 0.123, *N* = 87, *p* = 0.092) or women (τ = 0.118, *N* = 90, *p* = 0.101). Finally, the absolute difference of F0 was weakly positively associated with sociosexuality in men (τ = 0.139, *N* = 80, *p* = 0.069) but not in women (τ = 0.036, *N* = 84, *p* = 0.632). There were no significant correlations with the absolute difference between spoken and sung F0 range or VTL, rated attractiveness, and sociosexuality.

### The Effect of Targets’ Sex and Country on Voice Attractiveness Ratings From Speech and Singing

Test of between-subjects effects of the GLM model showed significant main effect of sex of targets on attractiveness rated both from speech (F = 13.84, df = 1, 157, *p* < 0.001, η_p_^2^ = 0.082) and singing (F = 36.48, df = 1, 157, *p* < 0.001, η_p_^2^ = 0.192). Estimated marginal means revealed that the voices of female participants were rated as more attractive based on both speech (mean rating = 3.89, SD = 0.65) and singing (mean rating = 3.82, SD = 0.73) than the voices of male participants (mean ratings = 3.48, SD = 0.66; and 3.11, SD = 0.72, respectively). There was no effect of country.

## Discussion

Using a cross-cultural sample of men and women, we have shown that speech and singing attractiveness are strongly correlated. We also found a strong correlation between the fundamental frequency (F0), F0 range, and vocal tract length (VTL) in both vocal displays. In men, low-pitched speech was rated as attractive and a similar trend was observed in singing. Furthermore, both vocal displays were invariably associated with body size (but not shape) and differently associated with sociosexuality. In women, both high-pitched singing and speaking voice predicted vocal attractiveness, and similarly to men, VTL as measured from singing and speech was differently associated with sociosexuality. Most results were invariant with respect to participants’ nationality, which indicates a degree of universality.

Our results partly support the hypothesis that speech and singing work as backup signals. They share many vocal parameters, such as fundamental frequency, its range and formant frequencies, which could lead to similar attractiveness ratings in both vocal displays (for similar results, see Isenstein, 2016). Both studied vocal displays thus covary in their production and perception and can transmit similar information to listeners. This is in line with previous studies which show that women’s cross-modal attractiveness or masculinity as rated from faces and spoken voices are intercorrelated, although no such correlation was found in men (Valentova et al., 2016; Pereira et al., 2019).

Nevertheless, we also found some features which are specific to the singing and speaking voice. For example, male speech attractiveness, but not singing attractiveness, is associated with higher sociosexuality (for similar results, see Hughes et al., 2004). The observed absence of association between singing attractiveness and male sociosexuality may suggest that singing voice is not part of the repertoire of short-term sexual strategy, at least in the two studied populations, which does not, however, exclude the possibility that it may be used to foster long-term relationships. Further, in line with previous studies, lower F0 in speech was directly connected to higher sociosexuality in men (e.g., Puts, 2005), while lower F0 in singing was connected to lower sociosexuality. Again, this could point to possible use of singing vocal display rather for committed long-term sexual strategy, which needs to be tested in future studies.

Further, although a high F0 in both speech and singing predicted vocal attractiveness in women, only low speech F0 was rated as attractive in men, although a similar non-significant trend appeared also in singing. This is in line with a study that found no difference in the attractiveness ratings of high- and low-pitched performances of famous singers (Neumann et al., 2008). Nevertheless, when analyzing the relative vocal parameters (difference in voice pitch between spoken and sung voice of the same person), we found that the singing voice of individuals who are capable of a higher pitch modulation is perceived as more attractive. In accordance with the handicap theory, individuals who can produce a larger difference between their spoken baseline and singing performance can thus benefit in terms of higher attractiveness and consequently potentially higher fitness. In line with this, men who modulated their voice pitch more had a tendency for higher sociosexuality, and men who like to sing more had more attractive voices. Both singing experience and higher capacity of voice modulation are thus linked to male attractiveness and sexuality.

Interestingly, in our study speech was on average rated as more attractive than singing. This can indicate that the standards for evaluation are higher in the singing domain, whereby singing abilities (e.g., singing in-tune), which are 40% heritable (Park et al., 2012), and were not tested in this study, may have influenced this difference. Nevertheless, another study found higher attractiveness ratings of singing than in speech in women and found no association between attractiveness ratings and singing quality (Isenstein, 2016). More studies are clearly needed to discern and determine the overall pattern.

We found that body weight was a strong positive predictor of both speech and singing attractiveness in men and a weak negative predictor of singing attractiveness in women (for similar results, see e.g., Sell et al., 2010; Xu et al., 2013; Šebesta et al., 2017). Weight also positively predicted VTL as estimated from speech in men, which is likewise in line with previous studies (for a review, see Pisanski et al., 2014). Some studies found differences in several vocal parameters (F0, voice pressure, perceptual voice quality) as a function of body weight, whereby heavier individuals have lower-pitched voices of more attractive perceptual quality (Barsties et al., 2013; Jost et al., 2018). The link between decrease in F0 and increase in body weight could be driven by hormonal factors, since for example in men, increased amount of fat tissue relates to lower testosterone levels (Zumoff et al., 1990; Tchernof et al., 1995). On the other hand, body weight may be due to not only body fat but also muscularity, which are both correlated with body size. Since the male body is composed relatively more by muscles than by fat tissue, one could speculate that vocal attractiveness provides a reliable cue specifically to muscularity, but future studies should assess the contribution of individual body components to vocal attractiveness. We also predicted a stronger association between body size and singing attractiveness but our results did not confirm this hypothesis. In humans, as in some songbirds (Hall et al., 2013), different vocal manifestations can thus serve as a cue to body size but not to body shape. This is in line with the finding that lower-pitched voice affects the perception of physical dominance (Puts et al., 2007).

Although women report that they like to sing more than men (Varella et al., 2010), and women and men both prefer sexual partners who demonstrate some music abilities (Kaufman et al., 2016), we found no association between singing or speaking voice attractiveness and sociosexuality or body indicators in women. This is contrary to previous studies (e.g., Hughes et al., 2004) which reported that women with attractive speaking voices had a lower waist-to-hip ratio, age of first sex, and a higher total number of sexual partners. Nevertheless, we found that shorter VTL measured from speech and longer VTL measured from singing predicted higher sociosexuality in women (for similar results in men, see Hodges-Simeon et al., 2011). This is comparable to our finding obtained for men when we analyzed the fundamental frequency. Generally speaking, individuals with sex-typical speech parameters and sex atypical singing parameters have higher sexual success (see, Bártová et al., 2019, for similar results on higher sociosexuality and gender non-conformity), which further supports the handicap hypothesis. Interestingly, there was no effect of the VTL on voice attractiveness and no effect of voice attractiveness on sociosexuality in women. Women’s tendency for sexual variety thus does not seem to be defined by how attractive they appear to the opposite sex. Access to sexual partners in individuals who display honest signals can be influenced by other mechanisms, such as intra-sexual competition (Varella et al., 2017; Ostrander et al., 2018).

This is the first study whose aim was to test the potential involvement of intersexual selection on different vocal displays on a cross-cultural sample of men and women (for intrasexual selection, see Raine et al., 2018; Šebesta et al., 2019). Although we used four different vocal recordings (standardized self-presentation, singing of “Happy Birthday,” and reading and singing of the national anthem), they do not represent the full range of human speech or singing. Standardized songs, such as “Happy Birthday,” are likely to limit pitch dynamics and range and thereby obscure or dampen the individual differences in pitch and voice modulation which might otherwise provide important cues to fitness.

Studies using different vocal recordings, such as spontaneous speech and singing, singing of more mating-relevant songs, or wordless singing, should be undertaken. This might be why some our predictions were not supported. It is for instance possible that a link between quality indicators and singing attractiveness becomes apparent in more demanding singing that involves complex rhythms, melody, or range (Charlton, 2014). The production of such demanding songs could be viewed as costly signaling and therefore serve as a more reliable indicator than the relatively undemanding songs employed in this study. Moreover, future studies should also perform more fine-tuned vocal analyses to compare both singing and speech (Šebesta et al., 2019).

It also ought to be taken into account that our samples in both countries were recruited from middle-class university student populations in the largest cities of both countries. They were thus not representative of the local populations and moreover compared only two countries. More cross-cultural comparisons are needed to test the generalizability potential of our current findings (see, Moshontz et al., 2018 for multi-lab psychological studies). Finally, as correlations between Czech and Brazilian raters were high, we pooled the ratings together, and did not analyze potential in-group and out-group effects, which might be addressed in future studies.

To conclude, we expected that singing would be a stronger indicator of individual body characteristics and sexuality than speech but our results show that cross-culturally, speech and singing seem to work rather in concert, i.e., as backup signals. Attractiveness of both singing and speaking voice is perceived in a similar way and is connected to a higher pitch in women and a lower pitch in men. Moreover, in men, speaking and singing both serve as similar cues to body indicators. On the other hand, the relation between speaking and singing voice and sociosexuality works in opposite ways in both men and women. Developmental pathways leading to sex-typical or atypical speaking and singing voice and sexuality should be addressed in future studies. In general, singing, together with other vocalizations, should be taken into account in evolutionary literature on voice production and perception.

## Ethics Statement

This study was carried out in accordance with the recommendations of the Charles University IRB with written informed consent from all subjects. All subjects gave written informed consent in accordance with the Declaration of Helsinki. The protocol was approved by the Charles University IRB (2011/07).

## Author Contributions

JV and JH developed the study concept and MV expanded it. JV, MV, FM, KP, LK, and PS collected the data. JV performed the analysis of F0 and F0 range of the vocal stimuli. PŠ performed the formant analyses during revisions of the manuscript. JV and PT performed the data analysis and interpretation jointly with MV and JH. JV and MV drafted the manuscript. PT and JH provided the critical revisions. JV, MV, JH, PŠ, and PT worked on the revised version of the manuscript. All authors approved the final version of the manuscript for submission.

## Conflict of Interest Statement

The authors declare that the research was conducted in the absence of any commercial or financial relationships that could be construed as a potential conflict of interest.

## References

[B1] Åkerlund L. Gramming P. (1994). Average loudness level, mean fundamental frequency, and subglottal pressure: comparison between female singers and nonsingers. *J. Voice* 8 263–270. 10.1016/s0892-1997(05)80298-x 7987429

[B2] Apicella C. L. Feinberg D. R. (2009). Voice pitch alters mate-choice-relevant perception in hunter – gatherers. *Proc. R. Soc. B* 276 1077–1082. 10.1098/rspb.2008.1542 19129125 PMC2679070

[B3] Apicella C. L. Feinberg D. R. Marlowe F. W. (2007). Voice pitch predicts reproductive success in male hunter-gatherers. *Biol. Lett.* 3 682–684. 10.1098/rsbl.2007.0410 17895219 PMC2391230

[B4] Babel M. McGuire G. King J. (2014). Towards a more nuanced view of vocal attractiveness. *PLoS One* 9:e88616. 10.1371/journal.pone.0088616 24586358 PMC3929563

[B5] Barsties B. Verfaillie R. Roy N. Maryn Y. (2013). Do body mass index and fat volume influence vocal quality, phonatory range, and aerodynamics in females? *Codas* 25 310–318. 24408481 10.1590/s2317-17822013000400003

[B6] Bártová K. Štěrbová Z. Varella M. A. C. Valentova J. V. (2019). Femininity in men and masculinity in women is positively related to sociosexuality. *Pers. Individ. Differ.* 152:109575 10.1016/j.paid.2019.109575

[B7] Boersma P. Weenink D. (2013). *Praat: Doing Phonetics by Computer [Computer Program], Version 5.3.42*. Available at: http://www.fon.hum.uva.nl/praat/ 10.1590/s2317-17822013000400003 (accessed July, 2019).

[B8] Borkowska B. Pawlowski B. (2011). Female voice frequency in the context of dominance and attractiveness perception. *Anim. Behav.* 82 55–59. 10.1016/j.anbehav.2011.03.024

[B9] Broesch T. L. Bryant G. A. (2015). Prosody in infant-directed speech is similar across western and traditional cultures. *J. Cogn. Dev.* 16 31–43. 10.1080/15248372.2013.833923

[B10] Bro-Jørgensen J. (2010). Dynamics of multiple signalling systems: animal communication in a world in flux. *Trends Ecol. Evol.* 25 292–300. 10.1016/j.tree.2009.11.003 20022401

[B11] Brown D. E. (1991). *Human Universals.* New York, NY: McGraw Hill.

[B12] Brown S. (2001). “The “musilanguage” model of music evolution,” in *The Origins of Music*, eds Wallin N. L. Merker B. Brown S., (Cambridge, MA: MIT Press), 271–300.

[B13] Brown S. (2017). A joint prosodic origin of language and music. *Front. Psychol.* 8:1894. 10.3389/fpsyg.2017.01894 29163276 PMC5666296

[B14] Bruckert L. Liénard J.-S. Lacroix A. Kreutzer M. Leboucher G. (2006). Women use voice parameters to assess men’s characteristics. *Proc. R. Soc. B Biol. Sci.* 273 83–89. 10.1098/rspb.2005.3265 16519239 PMC1560007

[B15] Busch S. L. (2013). *Beyond Singer vs. Non-singer in Singing, Health and Well-being: Development and Testing of the Singing Experience Scale.* Doctoral dissertation, Carleton University, Ottawa, ON.

[B16] Charlton B. D. (2014). Menstrual cycle phase alters women’s sexual preferences for composers of more complex music. *Proc. R. Soc. B* 281:20140403. 10.1098/rspb.2014.0403 24759864 PMC4043099

[B17] Christiner M. Reiterer S. M. (2013). Song and speech: examining the link between singing talent and speech imitation ability. *Front. Psychol.* 4:874. 10.3389/fpsyg.2013.00874 24319438 PMC3837232

[B18] Collins S. A. (2000). Men’s voices and women’s choices. *Anim. Behav.* 60 773–780. 11124875 10.1006/anbe.2000.1523

[B19] Collins S. A. Missing C. (2003). Vocal and visual attractiveness are related in women. *Anim. Behav.* 65 997–1004. 10.1006/anbe.2003.2123 23531807

[B20] Corretge R. (2019). *Praat Vocal Toolkit.* Available at: http://www.praatvocaltoolkit.com (accessed July, 2019).

[B21] Darwin C. (1871). *The Descent of Man and Selection in Relation to Sex.* London: J. Murray.

[B22] Dixson A. F. Halliwell G. East R. Wignarajah P. Anderson M. J. (2003). Masculine somatotype and hirsuteness as determinants of sexual attractiveness to women. *Arch. Sex. Behav.* 32 29–39. 10.1023/a:1021889228469 12597270

[B23] Dukes R. L. Bisel T. M. Borega K. N. Lobato E. A. Owens M. D. (2003). Expressions of love, sex, and hurt in popular songs: a content analysis of all-time greatest hits. *Soc. Sci. J.* 40 643–650. 10.1016/s0362-3319(03)00075-2

[B24] Evans S. Neave N. Wakelin D. (2006). Relationships between vocal characteristics and body size and shape in human males: an evolutionary explanation for a deep male voice. *Biol. Psychol.* 72 160–163. 10.1016/j.biopsycho.2005.09.003 16280195

[B25] Evans S. Neave N. Wakelin D. Hamilton C. (2008). The relationship between testosterone and vocal frequencies in human males. *Physiol. Behav.* 93 783–788. 10.1016/j.physbeh.2007.11.033 18155094

[B26] Feinberg D. R. Jones B. C. Little A. C. Burt D. M. Perrett D. I. (2005). Manipulations of fundamental and formant frequencies influence the attractiveness of human male voices. *Anim. Behav.* 69 561–568. 10.1016/j.anbehav.2004.06.012

[B27] Filippi P. (2016). Emotional and interactional prosody across animal communication systems: a comparative approach to the emergence of language. *Front. Psychol.* 7:1393. 10.3389/fpsyg.2016.01393 27733835 PMC5039945

[B28] Fitch W. T. (2005). The evolution of language: a comparative review. *Biol. Philos.* 20 193–203. 10.1007/s10539-005-5597-1

[B29] Fitch W. T. (2006). The biology and evolution of music: a comparative perspective. *Cognition* 100 173–215. 10.1016/j.cognition.2005.11.009 16412411

[B30] Foulkes P. Docherty G. J. Watt D. (2005). Phonological variation in child-directed speech. *Language* 81 177–206. 10.3109/02699206.2015.1115555 26828805

[B31] Fraccaro P. J. Jones B. C. Vukovic J. Smith F. G. Watkins C. D. Feinberg D. R. (2011). Experimental evidence that women speak in a higher voice pitch to men they find attractive. *J. Evol. Psychol.* 9 57–67. 10.1556/jep.9.2011.33.1

[B32] Garland H. Brown B. R. (1972). Face-saving as affected by subjects’ sex, audiences’ sex and audience expertise. *Sociometry* 35 280–289. 5033657

[B33] Hall M. L. Kingma S. A. Peters A. (2013). Male songbird indicates body size with low-pitched advertising songs. *PLoS One* 8:e56717. 10.1371/journal.pone.0056717 23437221 PMC3577745

[B34] Hodges-Simeon C. R. Gaulin S. J. Puts D. A. (2011). Voice correlates of mating success in men: examining “contests” versus “mate choice” modes of sexual selection. *Arch. Sex. Behav.* 40 551–557. 10.1007/s10508-010-9625-0 20369377

[B35] Hodges-Simeon C. R. Gurven M. Gaulin S. J. C. (2015). The low male voice is a costly signal of phenotypic quality among Bolivian adolescents. *Evol. Hum. Behav.* 36 294–302. 10.1016/j.evolhumbehav.2015.01.002

[B36] Hughes S. M. Dispenza F. Gallup G. G. (2004). Ratings of voice attractiveness predict sexual behavior and body configuration. *Evol. Hum. Behav.* 25 295–304. 10.1016/j.evolhumbehav.2004.06.001

[B37] Hughes S. M. Mogilski J. K. Harrison M. A. (2014). The perception and parameters of intentional voice manipulation. *J. Nonverbal. Behav.* 38 107–127. 10.1007/s10919-013-0163-z

[B38] Isenstein S. (2016). *Singing Voice Attractiveness.* Master thesis, McMaster University, Hamilton, ON.

[B39] Janik V. M. (2013). Cognitive skills in bottlenose dolphin communication. *Trends Cogn. Sci.* 17 157–159. 10.1016/j.tics.2013.02.005 23518158

[B40] Johnstone R. A. (1996). Multiple displays in animal communication: ‘backup signals’ and ‘multiple messages’. *Philos. Trans. R. Soc. B* 351 329–338. 10.1098/rstb.1996.0026

[B41] Jost L. Fuchs M. Loeffler M. Thiery J. Kratzsch J. Berger T. (2018). Associations of sex hormones and anthropometry with the speaking voice profile in the adult general population. *J. Voice* 32 261–272. 10.1016/j.jvoice.2017.06.011 28739331

[B42] Juslin P. N. Laukka P. (2003). Communication of emotions in vocal expression and music performance: different channels, same code? *Psychol. Bull.* 129 770–814. 10.1037/0033-2909.129.5.770 12956543

[B43] Kachel S. Radtke A. Skuk V. G. Zäske R. Simpson A. P. Steffens M. C. (2018). Investigating the common set of acoustic parameters in sexual orientation groups: a voice averaging approach. *PLoS One* 13:e0208686. 10.1371/journal.pone.0208686 30532156 PMC6287851

[B44] Kaufman S. B. Kozbelt A. Silvia P. Kaufman J. C. Ramesh S. Feist G. J. (2016). Who finds Bill Gates sexy? Creative mate preferences as a function of cognitive ability, personality, and creative achievement. *J. Creat. Behav.* 50 294–307. 10.1002/jocb.78

[B45] Klofstad C. A. Anderson R. C. Peters S. (2012). Sounds like a winner: voice pitch influences perception of leadership capacity in both men and women. *Proc. R. Soc. B Biol. Sci.* 279 2698–2704. 10.1098/rspb.2012.0311 22418254 PMC3350713

[B46] Leanderson R. Sundberg J. Von Euler C. (1987). Breathing muscle activity and subglottal pressure dynamics in singing and speech. *J. Voice* 1 258–261. 10.1016/s0892-1997(87)80009-7

[B47] Lehmann C. Welker L. Schiefenhövel W. (2009). Towards an ethology of song: a categorization of musical behaviour. *Music. Sci.* 13 321–338. 10.1177/1029864909013002141

[B48] Leongómez J. D. Binter J. Kubicová L. Stolařová P. Klapilová K. Havlíček J. (2014). Vocal modulation during courtship increases proceptivity even in naive listeners. *Evol. Hum. Behav.* 35 489–496. 10.1016/j.evolhumbehav.2014.06.008

[B49] Levitin D. J. (2008). *The World in Six Songs: How the Musical Brain Created Human Nature.* New York, NY: Penguin.

[B50] Little A. C. Jones B. C. Penton-Voak I. S. Burt D. M. Perrett D. I. (2002). Partnership status and the temporal context of relationships influence human female preferences for sexual dimorphism in male face shape. *Proc. R. Soc. B Biol. Sci.* 269 1095–1100. 10.1098/rspb.2002.1984 12061950 PMC1691012

[B51] Ma W. Fiveash A. Thompson W. F. (2019). Spontaneous emergence of language-like and music-like vocalizations from an artificial protolanguage. *Semiotica* 229 1–23. 10.1515/sem-2018-0139 [Epub ahead of print].

[B52] Mehr S. A. Singh M. York H. Glowacki L. Krasnow M. M. (2018). Form and function in human song. *Curr. Biol.* 28 356–368. 10.1016/j.cub.2017.12.042 29395919 PMC5805477

[B53] Mennen I. Schaeffler F. Docherty G. (2012). Cross-language differences in fundamental frequency range: a comparison of English and German. *J. Acoust. Soc. Am.* 131 2249–2260. 10.1121/1.3681950 22423720

[B54] Miller G. (2000). “Evolution of human music through sexual selection,” in *The Origins of Music*, eds Wallin N. L. Merker B. Brown S., (Cambridge MA: MIT Press), 329–360.

[B55] Mithen S. (2005). *The Singing Neanderthal.* London: Weidenfeld & Nicholson.

[B56] Moshontz H. Campbell L. Ebersole C. R. IJzerman H. Urry H. L. Forscher P. S. (2018). The psychological science accelerator: advancing psychology through a distributed collaborative network. *Adv. Meth. Pract. Psychol. Sci.* 1 501–515. 10.1177/2515245918797607 PMC6934079 31886452

[B57] Neumann K. Schunda P. Lehmann C. Euler H. A. (2008). Attractiveness of the high male speaking and singing voice. *Paper Presented at Conference Choice for the Voice of the British Voice Association*, London.

[B58] Ostrander G. M. Pipitone R. N. Shoup-Knox M. L. (2018). Interactions between observer and stimuli fertility status: endocrine and perceptual responses to intrasexual vocal fertility cues. *Horm. Behav.* 98 191–197. 10.1016/j.yhbeh.2017.12.008 29277698

[B59] Park H. Lee S. Kim H. J. Ju Y. S. Shin J. Y. Hong D. (2012). Comprehensive genomic analyses associate UGT8 variants with musical ability in a Mongolian population. *J. Med. Genet.* 49 747–752. 10.1136/jmedgenet-2012-101209 23118445 PMC3512346

[B60] Penke L. Asendorpf J. B. (2008). Beyond global sociosexual orientations: a more differentiated look at sociosexuality and its effects on courtship and romantic relationships. *J. Pers. Soc. Psychol.* 95 1113–1135. 10.1037/0022-3514.95.5.1113 18954197

[B61] Pereira K. J. da Silva C. S. A. Havlíček J. Kleisner K. Varella M. A. C. Pavlovič O. (2019). Femininity-masculinity and attractiveness – Associations between self-ratings, third-party ratings and objective measures. *Pers. Individ. Differ.* 147 166–171. 10.1016/j.paid.2019.04.033

[B62] Peretz I. Coltheart M. (2003). Modularity of music processing. *Nat. Neurosci.* 6 688–691. 10.1038/nn1083 12830160

[B63] Pisanski K. Cartei V. McGettigan C. Raine J. Reby D. (2016). Voice modulation: a window into the origins of human vocal control? *Trends Cogn. Sci.* 20 304–318. 10.1016/j.tics.2016.01.002 26857619

[B64] Pisanski K. Feinberg D. R. (2019). “Vocal attractiveness,” in *Oxford Handbook of Voice Perception*, eds Frühholz S. Belin P., (New York, NY: Oxford University Press), 607–625.

[B65] Pisanski K. Fraccaro P. J. Tigue C. C. O’Connor J. J. M. Röder S. Andrews P. W. (2014). Vocal indicators of body size in men and women: a meta-analysis. *Anim. Behav.* 95 89–99. 10.1016/j.anbehav.2014.06.011

[B66] Pisanski K. Oleszkiewicz A. Plachetka J. Gmiterek M. Reby D. (2018). Voice pitch modulation in human mate choice. *Proc. R. Soc. B* 285:20181634. 10.1098/rspb.2018.1634 30963886 PMC6304053

[B67] Puts D. A. (2005). Mating context and menstrual phase affect women’s preferences for male voice pitch. *Evol. Hum. Behav.* 26 388–397. 10.1016/j.evolhumbehav.2005.03.001

[B68] Puts D. A. (2010). Beauty and the beast: mechanisms of sexual selection in humans. *Evol. Hum. Behav.* 31 157–175. 10.1016/j.evolhumbehav.2010.02.005

[B69] Puts D. A. Barndt J. L. Welling L. L. Dawood K. Burriss R. P. (2011). Intrasexual competition among women: vocal femininity affects perceptions of attractiveness and flirtatiousness. *Pers. Indiv. Differ.* 50 111–115. 10.1016/j.paid.2010.09.011

[B70] Puts D. A. Gaulin S. J. C. Verdolini K. V. (2006). Dominance and the evolution of sexual dimorphism in human voice pitch. *Evol. Hum. Behav.* 27 283–296. 10.1016/j.evolhumbehav.2005.11.003

[B71] Puts D. A. Hill A. K. Bailey D. H. Walker R. S. Rendall D. Wheatley J. R. (2016). Sexual selection on male vocal fundamental frequency in humans and other anthropoids. *Proc. R. Soc. B Biol. Sci.* 283:20152830. 10.1098/rspb.2015.2830 27122553 PMC4855375

[B72] Puts D. A. Hodges C. R. Cárdenas R. A. Gaulin S. J. C. (2007). Men’s voices as dominance signals: vocal fundamental and formant frequencies influence dominance attributions among men. *Evol. Hum. Behav.* 28 340–344. 10.1016/j.evolhumbehav.2007.05.002

[B73] Raine J. Pisanski K. Bond R. Simner J. Reby D. (2019). Human roars communicate upper-body strength more effectively than do screams or aggressive and distressed speech. *PLoS One* 14:e0213034. 10.1371/journal.pone.0213034 30830931 PMC6398857

[B74] Raine J. Pisanski K. Oleszkiewicz A. Simner J. Reby D. (2018). Human listeners can accurately judge strength and height relative to self from aggressive roars and speech. *iScience* 4 273–280. 10.1016/j.isci.2018.05.002 30240746 PMC6146593

[B75] Rowe M. L. (2012). A longitudinal investigation of the role of quantity and quality of child-directed speech in vocabulary development. *Child Dev.* 83 1762–1774. 10.1111/j.1467-8624.2012.01805.x 22716950 PMC3440540

[B76] Savage P. E. Tierney A. T. Patel A. D. (2017). Global music recordings support the motor constraint hypothesis for human and avian song contour. *Music Percept.* 34 327–334. 10.1525/mp.2017.34.3.327

[B77] Saxton T. K. Mackey L. L. McCarty K. Neave N. (2015). A lover or a fighter? Opposing sexual selection pressures on men’s vocal pitch and facial hair. *Behav. Ecol.* 27 512–519. 10.1093/beheco/arv178 27004013 PMC4797380

[B78] Searcy W. A. Andersson M. (1986). Sexual selection and the evolution of song. *Ann. Rev. Ecol. Sys.* 17 507–533.

[B79] Šebesta P. Kleisner K. Tureček P. Kočnar T. Akoko R. M. Třebický V. (2017). Voices of Africa: acoustic predictors of human male vocal attractiveness. *Anim. Behav.* 127 205–211. 10.1016/j.anbehav.2017.03.014

[B80] Šebesta P. Třebický V. Fialová J. Havlíček J. (2019). Roar of a champion: loudness and voice pitch predict perceived fighting ability but not success in MMA fighters. *Front. Psychol.* 10:859. 10.3389/fpsyg.2019.00859 31114519 PMC6502904

[B81] Sell A. Bryant G. A. Cosmides L. Tooby J. Sznycer D. Von Rueden C. (2010). Adaptations in humans for assessing physical strength from the voice. *Proc. R. Soc. B* 277 3509–3518. 10.1098/rspb.2010.0769 20554544 PMC2982226

[B82] Shirazi T. N. Puts D. A. Escasa-Dorne M. J. (2018). Filipino women’s preferences for male voice pitch: intra-individual, life history, and hormonal predictors. *Adapt. Hum. Behav. Physiol.* 4 188–206. 10.1007/s40750-018-0087-2

[B83] Slobodchikoff C. N. Kiriazis J. Fischer C. Creef E. (1991). Semantic information distinguishing individual predators in the alarm calls of Gunnison’s prairie dogs. *Anim. Behav.* 42 713–719. 10.1016/s0003-3472(05)80117-4 11011104

[B84] Smith E. A. Bird R. L. B. (2000). Turtle hunting and tombstone opening: public generosity as costly signaling. *Evol. Hum. Behav.* 21 245–261. 10.1016/S1090-5138(00)00031-3 10899477

[B85] Spinelli M. Fasolo M. Mesman J. (2017). Does prosody make the difference? A meta-analysis on relations between prosodic aspects of infant-directed speech and infant outcomes. *Devel. Rev.* 44 1–18. 10.1016/j.dr.2016.12.001

[B86] Stulp G. Buunk A. P. Pollet T. V. Nettle D. Verhulst S. (2013). Are human mating preferences with respect to height reflected in actual pairings? *PLoS One* 8:e54186. 10.1371/journal.pone.0054186 23342102 PMC3546926

[B87] Sundberg J. (1977). The acoustics of the singing voice. *Sci. Am.* 236 82–91. 10.1038/scientificamerican0377-82 841298

[B88] Sundberg J. (2003). Research on the singing voice in retrospect. *TMH QPSR* 45 11–22.

[B89] Sundberg J. (2018). “The singing voice,” in *The Oxford Handbook of Voice Perception*, eds Fruhholz S. Belin P., (Oxford: Oxford University Press), 117–142.

[B90] Tchernof A. Després J. P. Bélanger A. Dupont A. Prud’homme D. Moorjani S. (1995). Reduced testosterone and adrenal C19 steroid levels in obese men. *Metabolism* 44 513–519. 10.1016/0026-0495(95)90060-8 7723675

[B91] Trehub S. E. Plantinga J. Brcic J. (2009). Infants detect cross-modal cues to identity in speech and singing. *Ann. N. Y. Acad. Sci.* 1169 508–511. 10.1111/j.1749-6632.2009.04851.x 19673832

[B92] Valentová J. Roberts S. C. Havlíček J. (2013). Preferences for facial and vocal masculinity in homosexual men: the role of relationship status, sexual restrictiveness, and self-perceived masculinity. *Perception* 42 187–197. 10.1068/p6909 23700957

[B93] Valentova J. V. Bártová K. Štěrbová Z. Varella M. A. C. (2016). Preferred and actual relative height are related to sex, sexual orientation, and dominance: evidence from Czech Republic and Brazil. *Pers. Indiv. Differ.* 100 145–150. 10.1016/j.paid.2016.01.049

[B94] Valentova J. V. Bártová K. Štčrbová Z. Varella M. A. C. (2017a). Influence of sexual orientation, population, homogamy, and imprinting-like effect on preferences and choices for female buttock size, breast size and shape, and WHR. *Pers. Indiv. Differ.* 104 313–319. 10.1016/j.paid.2016.08.005

[B95] Valentova J. V. Havlíček J. (2013). Perceived sexual orientation based on vocal and facial stimuli is linked to self-rated sexual orientation in Czech men. *PLoS One* 8:e82417. 10.1371/journal.pone.0082417 24358180 PMC3864997

[B96] Valentova J. V. Stulp G. Třebický V. Havlíček J. (2014). Preferred and actual relative height among homosexual male partners vary with preferred dominance and sex role. *PLoS One* 9:e86534. 10.1371/journal.pone.0086534 24466136 PMC3899263

[B97] Valentova J. V. Varella M. A. C. Bártová K. Štěrbová Z. Dixson B. J. W. (2017b). Mate preferences and choices for facial and body hair in heterosexual women and homosexual men: influence of sex, population, homogamy, and imprinting-like effect. *Evol. Hum. Behav.* 38 241–248. 10.1016/j.evolhumbehav.2016.10.007

[B98] Valentova J. V. Varella M. A. C. Havlíček J. Kleisner K. (2017c). Positive association between vocal and facial attractiveness in women but not in men: a cross-cultural study. *Behav. Proc.* 135 95–100. 10.1016/j.beproc.2016.12.005 27986472

[B99] Varella M. A. C. Ferreira J. H. B. P. Cosentino L. A. M. Ottoni E. Bussab V. S. R. (2010). Sex differences in aspects of musicality in a Brazilian sample: adaptative hypotheses. *Cogn. Music. Arts* 4 10–16.

[B100] Varella M. A. C. Valentova J. V. Fernández A. M. (2017). “Evolution of artistic and aesthetic propensities through female competitive ornamentation,” in *The Oxford Handbook of Women and Competition*, ed. Fisher M., (New York, NY: Oxford University Press), 757–783.

[B101] Varella M. A. C. Valentova J. V. Pereira K. J. Bussab V. S. R. (2014). Promiscuity is related to masculine and feminine body traits in both men and women: evidence from Brazilian and Czech samples. *Behav. Process.* 109 34–39. 10.1016/j.beproc.2014.07.010 25093932

[B102] Vukovic J. Feinberg D. R. DeBruine L. Smith F. G. Jones B. C. (2010). Women’s voice pitch is negatively correlated with health risk factors. *J. Evol. Psychol.* 8 217–225. 10.1556/JEP.8.2010.3.2

[B103] Wapnick J. Darrow A. A. Kovacs J. Dalrymple L. (1997). Effects of physical attractiveness on evaluation of vocal performance. *J. Res. Music Educ.* 45 470–479. 10.2307/3345540

[B104] Welch G. F. (2006). “Singing and vocal development,” in *The Child as Musician: A Handbook of Musical Development*, ed. McPherson G., (New York, NY: Oxford University Press), 311–329.

[B105] Weninger F. Wöllmer M. Schuller B. (2011). “Automatic assessment of singer traits in popular music: Gender, age, height and race,” in *Proceedings of the 12th International Society for Music Information Retrieval Conference*, Miami, FL.

[B106] White J. Lorenz H. Perilloux C. Lee A. (2018). Creative casanovas: mating strategy predicts using—but not preferring—atypical flirting tactics. *Evol. Psych. Sci.* 4 443–455. 10.1007/s40806-018-0155-7

[B107] Xu Y. Lee A. Wu W. L. Liu X. Birkholz P. (2013). Human vocal attractiveness as signaled by body size projection. *PLoS One* 8:e62397. 10.1371/journal.pone.0062397 23638065 PMC3634748

[B108] Zarate J. M. (2013). The neural control of singing. *Front. Hum. Neurosci.* 7:237. 10.3389/fnhum.2013.00237 23761746 PMC3669747

[B109] Zatorre R. J. Baum S. R. (2012). Musical melody and speech intonation: singing a different tune. *PLoS Biol.* 10:e1001372. 10.1371/journal.pbio.1001372 22859909 PMC3409119

[B110] Zumoff B. Strain G. W. Miller L. K. Rosner W. Senie R. Seres D. S. (1990). Plasma free and non-sex-hormone-binding-globulin bound testosterone are decreased in obese men in proportion to their degree of obesity. *J. Clin. Endocrinol. Metab.* 71 929–931. 10.1210/jcem-71-4-929 2401718

