# Supplementary material for: Vocal Parameters of Speech and Singing Covary and Are Related to Vocal Attractiveness, Body Measures, and Sociosexuality: A Cross-Cultural Study
Source: Front Psychol. 2019 Oct 22;10:2029. doi: 10.3389/fpsyg.2019.02029 (PMC6817625; doi:10.3389/fpsyg.2019.02029)
Supplement: Supplementary file 1 [file Table_1.DOC]

**Supplementary material**

**Additional results, correlations between ratings, correlations between vocal measurements, inter-rater agreement**

| **Table S1.** Pearson's product moment correlations between average attractiveness ratings | | | | | | | | |
| --- | --- | --- | --- | --- | --- | --- | --- | --- |
|  | HB BR | HB CZ | IN BR | IN CZ | SA BR | SA CZ | TA BR | TA CZ |
| HB BR | 1 | 0.77 | 0.71 | 0.55 | 0.74 | 0.65 | 0.66 | 0.51 |
| HB CZ | 0.77 | 1 | 0.57 | 0.6 | 0.64 | 0.76 | 0.51 | 0.6 |
| IN BR | 0.71 | 0.57 | 1 | 0.66 | 0.6 | 0.47 | 0.76 | 0.53 |
| IN CZ | 0.55 | 0.6 | 0.66 | 1 | 0.43 | 0.53 | 0.56 | 0.68 |
| SA BR | 0.74 | 0.64 | 0.6 | 0.43 | 1 | 0.7 | 0.58 | 0.39 |
| SA CZ | 0.65 | 0.76 | 0.47 | 0.53 | 0.7 | 1 | 0.5 | 0.61 |
| TA BR | 0.66 | 0.51 | 0.76 | 0.56 | 0.58 | 0.5 | 1 | 0.64 |
| TA CZ | 0.51 | 0.6 | 0.53 | 0.68 | 0.39 | 0.61 | 0.64 | 1 |
| First pair of letters corresponds to the recording (HB=Happy birthday, IN=Introduction, SA=singing anthem, TA=talking anthem), second pair of letters indicates the nationality of raters (BR=Brazilian, CZ=Czech) | | | | | | | | |

| **Table S2.** Pearson's product moment correlations between acoustic measures of recordings in Hz (notice the strong collinearity between recording's F0 and range) | | | | | | | | |
| --- | --- | --- | --- | --- | --- | --- | --- | --- |
|  | HB F0 | HB range | IN F0 | IN range | SA F0 | SA range | TA F0 | TA range |
| HB F0 | 1 | 0.79 | 0.93 | 0.52 | 0.97 | 0.81 | 0.94 | 0.53 |
| HB range | 0.79 | 1 | 0.74 | 0.44 | 0.76 | 0.69 | 0.74 | 0.49 |
| IN F0 | 0.93 | 0.74 | 1 | 0.6 | 0.93 | 0.78 | 0.98 | 0.52 |
| IN range | 0.52 | 0.44 | 0.6 | 1 | 0.5 | 0.5 | 0.57 | 0.43 |
| SA F0 | 0.97 | 0.76 | 0.93 | 0.5 | 1 | 0.81 | 0.93 | 0.54 |
| SA range | 0.81 | 0.69 | 0.78 | 0.5 | 0.81 | 1 | 0.78 | 0.5 |
| TA F0 | 0.94 | 0.74 | 0.98 | 0.57 | 0.93 | 0.78 | 1 | 0.53 |
| TA range | 0.53 | 0.49 | 0.52 | 0.43 | 0.54 | 0.5 | 0.53 | 1 |
| HB=Happy birthday, IN=Introduction, SA=singing anthem, TA=talking anthem | | | | | | | | |

| **Table S3.** Pearson's product moment correlations between acoustic measures of recordings in semitones | | | | | | | | |
| --- | --- | --- | --- | --- | --- | --- | --- | --- |
|  | HB F0 | HB range | IN F0 | IN range | SA F0 | SA range | TA F0 | TA range |
| HB F0 | 1 | 0.24 | 0.95 | 0.27 | 0.97 | 0.26 | 0.95 | 0.22 |
| HB range | 0.24 | 1 | 0.2 | 0.18 | 0.2 | 0.26 | 0.19 | 0.12 |
| IN F0 | 0.95 | 0.2 | 1 | 0.33 | 0.95 | 0.21 | 0.98 | 0.2 |
| IN range | 0.27 | 0.18 | 0.33 | 1 | 0.26 | 0.25 | 0.3 | 0.21 |
| SA F0 | 0.97 | 0.2 | 0.95 | 0.26 | 1 | 0.24 | 0.94 | 0.24 |
| SA range | 0.26 | 0.26 | 0.21 | 0.25 | 0.24 | 1 | 0.21 | 0.17 |
| TA F0 | 0.95 | 0.19 | 0.98 | 0.3 | 0.94 | 0.21 | 1 | 0.21 |
| TA range | 0.22 | 0.12 | 0.2 | 0.21 | 0.24 | 0.17 | 0.21 | 1 |
| HB=Happy birthday, IN=Introduction, SA=singing anthem, TA=talking anthem | | | | | | | | |

| **Table S4.** Inter-rater agreement (Cronbach’s α) in all recording×rater sets (notice that Cronbach’s α in highest if both subsamples of raters are combined, treating subsamples of raters separately does not give us any new information) | | | | | | | | | | | | | |
| --- | --- | --- | --- | --- | --- | --- | --- | --- | --- | --- | --- | --- | --- |
|  | recording | Happy birthday | | | Introduction | | | Singing anthem | | | Talking anthem | | |
|  | raters | BR | CZ | both | BR | CZ | both | BR | CZ | both | BR | CZ | both |
| target | BR F | 0.89 | 0.9 | 0.94 | 0.86 | 0.8 | 0.89 | 0.86 | 0.79 | 0.88 | 0.88 | 0.88 | 0.92 |
|  | BR M | 0.89 | 0.9 | 0.94 | 0.91 | 0.87 | 0.93 | 0.86 | 0.87 | 0.92 | 0.89 | 0.89 | 0.93 |
|  | CZ F | 0.93 | 0.94 | 0.96 | 0.94 | 0.95 | 0.97 | 0.94 | 0.93 | 0.97 | 0.92 | 0.92 | 0.95 |
|  | CZ M | 0.92 | 0.94 | 0.96 | 0.86 | 0.93 | 0.94 | 0.89 | 0.93 | 0.95 | 0.82 | 0.93 | 0.93 |
| Nationality of both targets and raters is indicated (BR=Brazilian, CZ=Czech), Sex of targets is indicated (F=female, M=male), raters are always of the opposite sex then target voice recordings | | | | | | | | | | | | | |

**Table S5.** Mean fundamental frequency (F0) and range of fundamental frequency (F0 range) (in Hz) in men and women

|  | Men | | | Women | | |
| --- | --- | --- | --- | --- | --- | --- |
|  | Brazilian (N = 42) | Czech (N = 35) | Total  (N = 77) | Brazilian  (N = 45) | Czech  (N = 36) | Total  (N = 81) |
| Mean F0 – speech (SD) | 123.40 (15.69) | 119.89 (14.09) | 121.80 (14.99) | 205.17 (16.06) | 207.92 (18.14) | 206.39 (16.97) |
| Mean F0 – singing (SD) | 141.19 (21.87) | 135.81 (17.77) | 138.75 (20.16) | 240.56 (30.08) | 247.08 (29.84) | 243.46 (29.97) |
| Mean F0 range – speech (SD) | 89.37 (25.27) | 118.83 (29.08) | 102.76 (30.67) | 174.33 (65.78) | 230.12 (47.58) | 199.12 (64.41) |
| Mean F0 range – singing (SD) | 122.10 (31.43) | 118.83 (29.08) | 120.61 (30.23) | 215.77 (49.65) | 230.11 (47.58) | 222.15 (48.97) |

**Full model estimations**

| **Table S6.** Estimation of the full structural model for male participants | | | | | | | | | | |
| --- | --- | --- | --- | --- | --- | --- | --- | --- | --- | --- |
| Relationship |  |  | est. | SE | z | p (A) | p (R) | CI lower | CI upper | std. |
| WSR | ~~ | height | 0.01 | 0.042 | 0.23 | 0.818 | 0.82 | -0.072 | 0.091 | 0.027 |
| WSR | ~~ | weight | 0.292 | 0.073 | 3.977 | 0 | 0 | 0.148 | 0.435 | 0.526 |
| WSR | ~~ | age | -0.003 | 0.019 | -0.171 | 0.864 | 0.855 | -0.041 | 0.034 | -0.02 |
| height | ~~ | weight | 39.111 | 9.803 | 3.99 | 0 | 0 | 19.898 | 58.324 | 0.528 |
| height | ~~ | age | -1.607 | 2.561 | -0.627 | 0.53 | 0.551 | -6.627 | 3.413 | -0.074 |
| weight | ~~ | age | 0.045 | 3.976 | 0.011 | 0.991 | 0.983 | -7.747 | 7.837 | 0.001 |
| sing F0 | ~ | WSR | 4.032 | 6.698 | 0.602 | 0.547 | 0.481 | -9.095 | 17.159 | 0.087 |
| sing F0 | ~ | height | -0.043 | 0.05 | -0.848 | 0.397 | 0.3 | -0.141 | 0.056 | -0.123 |
| sing F0 | ~ | weight | 0.004 | 0.038 | 0.096 | 0.923 | 0.902 | -0.071 | 0.078 | 0.016 |
| sing F0 | ~ | age | 0.057 | 0.088 | 0.646 | 0.518 | 0.533 | -0.115 | 0.228 | 0.075 |
| sing range | ~ | WSR | -4.624 | 6.925 | -0.668 | 0.504 | 0.419 | -18.196 | 8.949 | -0.098 |
| sing range | ~ | height | 0.008 | 0.052 | 0.154 | 0.878 | 0.84 | -0.094 | 0.11 | 0.023 |
| sing range | ~ | weight | 0.021 | 0.039 | 0.546 | 0.585 | 0.429 | -0.056 | 0.098 | 0.094 |
| sing range | ~ | age | -0.021 | 0.091 | -0.237 | 0.813 | 0.813 | -0.199 | 0.156 | -0.028 |
| talk F0 | ~ | WSR | 2.645 | 5.542 | 0.477 | 0.633 | 0.56 | -8.216 | 13.507 | 0.069 |
| talk F0 | ~ | height | -0.057 | 0.042 | -1.356 | 0.175 | 0.105 | -0.138 | 0.025 | -0.196 |
| talk F0 | ~ | weight | -0.001 | 0.031 | -0.042 | 0.966 | 0.941 | -0.063 | 0.06 | -0.007 |
| talk F0 | ~ | age | 0.02 | 0.072 | 0.27 | 0.787 | 0.791 | -0.122 | 0.162 | 0.031 |
| talk range | ~ | WSR | -3.541 | 7.601 | -0.466 | 0.641 | 0.593 | -18.438 | 11.356 | -0.068 |
| talk range | ~ | height | 0.043 | 0.057 | 0.757 | 0.449 | 0.363 | -0.069 | 0.155 | 0.11 |
| talk range | ~ | weight | -0.026 | 0.043 | -0.611 | 0.541 | 0.403 | -0.111 | 0.058 | -0.104 |
| talk range | ~ | age | 0.046 | 0.099 | 0.463 | 0.643 | 0.653 | -0.149 | 0.241 | 0.054 |
| sing ATTR | ~ | sing F0 | -0.014 | 0.033 | -0.428 | 0.669 | 0.713 | -0.078 | 0.05 | -0.047 |
| sing ATTR | ~ | sing range | 0.065 | 0.032 | 2.045 | 0.041 | 0.068 | 0.003 | 0.127 | 0.221 |
| sing ATTR | ~ | WSR | -2.16 | 1.882 | -1.148 | 0.251 | 0.209 | -5.849 | 1.529 | -0.156 |
| sing ATTR | ~ | height | -0.015 | 0.014 | -1.055 | 0.291 | 0.238 | -0.043 | 0.013 | -0.144 |
| sing ATTR | ~ | weight | 0.029 | 0.011 | 2.723 | 0.006 | 0 | 0.008 | 0.05 | 0.434 |
| sing ATTR | ~ | age | -0.007 | 0.025 | -0.297 | 0.766 | 0.799 | -0.055 | 0.041 | -0.032 |
| talk ATTR | ~ | talk F0 | -0.123 | 0.032 | -3.843 | 0 | 0.001 | -0.186 | -0.06 | -0.357 |
| talk ATTR | ~ | talk range | 0.067 | 0.023 | 2.873 | 0.004 | 0.025 | 0.021 | 0.113 | 0.264 |
| talk ATTR | ~ | WSR | -1.377 | 1.518 | -0.907 | 0.364 | 0.369 | -4.353 | 1.598 | -0.104 |
| talk ATTR | ~ | height | -0.003 | 0.012 | -0.219 | 0.826 | 0.833 | -0.025 | 0.02 | -0.026 |
| talk ATTR | ~ | weight | 0.026 | 0.009 | 2.969 | 0.003 | 0 | 0.009 | 0.042 | 0.4 |
| talk ATTR | ~ | age | -0.057 | 0.02 | -2.866 | 0.004 | 0.028 | -0.096 | -0.018 | -0.262 |
| SOI total | ~ | sing ATTR | -1.9 | 2.225 | -0.854 | 0.393 | 0.487 | -6.261 | 2.462 | -0.073 |
| SOI total | ~ | sing F0 | 2.43 | 0.623 | 3.902 | 0 | 0.002 | 1.21 | 3.651 | 0.311 |
| SOI total | ~ | sing range | -0.629 | 0.619 | -1.017 | 0.309 | 0.428 | -1.841 | 0.583 | -0.083 |
| SOI total | ~ | talk ATTR | 6.302 | 2.752 | 2.29 | 0.022 | 0.033 | 0.908 | 11.695 | 0.231 |
| SOI total | ~ | talk F0 | -3.971 | 0.824 | -4.819 | 0 | 0 | -5.587 | -2.356 | -0.424 |
| SOI total | ~ | talk range | 1.143 | 0.578 | 1.976 | 0.048 | 0.115 | 0.01 | 2.276 | 0.166 |
| SOI total | ~ | WSR | -53.91 | 36.413 | -1.481 | 0.139 | 0.152 | -125.281 | 17.454 | -0.149 |
| SOI total | ~ | height | -0.504 | 0.276 | -1.83 | 0.067 | 0.072 | -1.045 | 0.036 | -0.187 |
| SOI total | ~ | weight | 0.289 | 0.224 | 1.289 | 0.197 | 0.113 | -0.15 | 0.728 | 0.166 |
| SOI total | ~ | age | 1.895 | 0.493 | 3.843 | 0 | 0.001 | 0.929 | 2.862 | 0.322 |
|  |  |  |  |  |  |  |  |  |  |  |
| ~~ indicates correlation, ~ regression coefficient, est.= estimated parameter, SE = standard error, p (A) = probability of the relationship being insignificant (standard lavaan output), p (R)= robust p value from permutation test, CI = 95% confidence interval of estimated parameter, std. = standardized parameter value, WSR=waist-to-shoulder ratio, ATTR = attractiveness, F0 = average fundamental frequency   | **Table S7.** Estimation of the full structural model for female participants | | | | | | | | | | | | --- | --- | --- | --- | --- | --- | --- | --- | --- | --- | --- | | Relationship |  |  | est. | SE | z | p (A) | p (R) | CI lower | CI upper | std. | | WHR | ~~ | height | 13.75 | 7.519 | 1.829 | 0.067 | 0.128 | -0.987 | 28.488 | 0.21 | | WHR | ~~ | weight | -0.193 | 13.57 | -0.014 | 0.989 | 0.84 | -26.789 | 26.404 | -0.002 | | WHR | ~~ | age | 7.167 | 5.157 | 1.39 | 0.165 | 0.291 | -2.941 | 17.275 | 0.158 | | height | ~~ | weight | 31.012 | 8.141 | 3.81 | 0 | 0 | 15.056 | 46.967 | 0.474 | | height | ~~ | age | -0.344 | 2.761 | -0.124 | 0.901 | 0.903 | -5.755 | 5.068 | -0.014 | | weight | ~~ | age | 2.18 | 5.097 | 0.428 | 0.669 | 0.661 | -7.811 | 12.17 | 0.048 | | sing F0 | ~ | WHR | 0.014 | 0.021 | 0.654 | 0.513 | 0.434 | -0.028 | 0.055 | 0.074 | | sing F0 | ~ | height | -0.018 | 0.044 | -0.401 | 0.688 | 0.656 | -0.104 | 0.068 | -0.051 | | sing F0 | ~ | weight | -0.043 | 0.023 | -1.837 | 0.066 | 0.052 | -0.088 | 0.003 | -0.229 | | sing F0 | ~ | age | -0.01 | 0.055 | -0.179 | 0.858 | 0.862 | -0.117 | 0.098 | -0.02 | | sing range | ~ | WHR | -0.006 | 0.032 | -0.177 | 0.859 | 0.976 | -0.069 | 0.057 | -0.02 | | sing range | ~ | height | 0.084 | 0.067 | 1.257 | 0.209 | 0.161 | -0.047 | 0.214 | 0.162 | | sing range | ~ | weight | -0.061 | 0.035 | -1.734 | 0.083 | 0.053 | -0.13 | 0.008 | -0.218 | | sing range | ~ | age | -0.078 | 0.083 | -0.935 | 0.35 | 0.368 | -0.241 | 0.085 | -0.104 | | talk F0 | ~ | WHR | -0.001 | 0.014 | -0.095 | 0.924 | 0.862 | -0.029 | 0.027 | -0.011 | | talk F0 | ~ | height | -0.035 | 0.03 | -1.191 | 0.234 | 0.187 | -0.093 | 0.023 | -0.152 | | talk F0 | ~ | weight | -0.012 | 0.016 | -0.737 | 0.461 | 0.431 | -0.042 | 0.019 | -0.092 | | talk F0 | ~ | age | -0.052 | 0.037 | -1.395 | 0.163 | 0.187 | -0.124 | 0.021 | -0.154 | | talk range | ~ | WHR | -0.056 | 0.041 | -1.368 | 0.171 | 0.225 | -0.136 | 0.024 | -0.155 | | talk range | ~ | height | -0.133 | 0.085 | -1.569 | 0.117 | 0.082 | -0.298 | 0.033 | -0.2 | | talk range | ~ | weight | 0.028 | 0.045 | 0.62 | 0.536 | 0.506 | -0.06 | 0.116 | 0.077 | | talk range | ~ | age | 0.007 | 0.106 | 0.07 | 0.945 | 0.948 | -0.2 | 0.215 | 0.008 | | sing ATTR | ~ | sing F0 | 0.169 | 0.03 | 5.542 | 0 | 0 | 0.109 | 0.229 | 0.516 | | sing ATTR | ~ | sing range | -0.035 | 0.02 | -1.768 | 0.077 | 0.166 | -0.075 | 0.004 | -0.163 | | sing ATTR | ~ | WHR | 0.001 | 0.006 | 0.253 | 0.8 | 0.835 | -0.01 | 0.013 | 0.024 | | sing ATTR | ~ | height | -0.005 | 0.012 | -0.386 | 0.699 | 0.738 | -0.028 | 0.019 | -0.041 | | sing ATTR | ~ | weight | -0.01 | 0.007 | -1.476 | 0.14 | 0.187 | -0.022 | 0.003 | -0.158 | | sing ATTR | ~ | age | -0.008 | 0.015 | -0.505 | 0.614 | 0.694 | -0.037 | 0.022 | -0.046 | | talk ATTR | ~ | talk F0 | 0.183 | 0.046 | 3.993 | 0 | 0.001 | 0.093 | 0.273 | 0.403 | | talk ATTR | ~ | talk range | 0.006 | 0.016 | 0.389 | 0.697 | 0.749 | -0.025 | 0.038 | 0.039 | | talk ATTR | ~ | WHR | 0.008 | 0.006 | 1.34 | 0.18 | 0.207 | -0.004 | 0.019 | 0.138 | | talk ATTR | ~ | height | -0.01 | 0.012 | -0.8 | 0.424 | 0.43 | -0.034 | 0.014 | -0.094 | | talk ATTR | ~ | weight | -0.008 | 0.006 | -1.253 | 0.21 | 0.232 | -0.021 | 0.005 | -0.141 | | talk ATTR | ~ | age | -0.005 | 0.015 | -0.305 | 0.76 | 0.81 | -0.035 | 0.025 | -0.031 | | SOI total | ~ | sing ATTR | -3.093 | 2.588 | -1.195 | 0.232 | 0.193 | -8.164 | 1.979 | -0.156 | | SOI total | ~ | sing F0 | 0.752 | 0.826 | 0.911 | 0.363 | 0.332 | -0.866 | 2.37 | 0.116 | | SOI total | ~ | sing range | 0.913 | 0.47 | 1.94 | 0.052 | 0.081 | -0.009 | 1.835 | 0.212 | | SOI total | ~ | talk ATTR | 3.418 | 2.543 | 1.344 | 0.179 | 0.177 | -1.566 | 8.402 | 0.162 | | SOI total | ~ | talk F0 | -1.743 | 1.137 | -1.533 | 0.125 | 0.13 | -3.971 | 0.486 | -0.182 | | SOI total | ~ | talk range | 0.356 | 0.364 | 0.98 | 0.327 | 0.382 | -0.356 | 1.069 | 0.106 | | SOI total | ~ | WHR | 0.029 | 0.135 | 0.215 | 0.83 | 0.804 | -0.236 | 0.294 | 0.024 | | SOI total | ~ | height | 0.327 | 0.284 | 1.153 | 0.249 | 0.223 | -0.229 | 0.884 | 0.147 | | SOI total | ~ | weight | -0.023 | 0.155 | -0.149 | 0.882 | 0.895 | -0.326 | 0.28 | -0.019 | | SOI total | ~ | age | 0.075 | 0.349 | 0.214 | 0.831 | 0.826 | -0.609 | 0.758 | 0.023 | | ~~ indicates correlation, ~ regression coefficient, est.= estimated parameter, SE = standard error, p (A) = probability of the relationship being insignificant (standard lavaan output), p (R)= robust p value from permutation test, CI = 95% confidence interval of estimated parameter, std. = standardized parameter value, WHR=waist-to-hip ratio, ATTR = attractiveness, F0 = average fundamental frequency | | | | | | | | | | | | | | | | | | | | | |

**Jackknife estimates and ranges of robust p values and standardized coefficients yielded by leaving out one observation at a time**

| **Table S8.** Jackknife estimations of robust p values and standardized coefficients in male sample | | | | | | | | | | | | |
| --- | --- | --- | --- | --- | --- | --- | --- | --- | --- | --- | --- | --- |
| Relationship |  |  | p (R) | JK | SD | min. | max. | std. | JK | SD | min. | max. |
| WSR | ~~ | height | 0.82 | 0.815 | 0.082 | 0.532 | 0.996 | 0.027 | 0.027 | 0.015 | -0.02 | 0.076 |
| WSR | ~~ | weight | 0 | 0 | 0 | 0 | 0 | 0.526 | 0.526 | 0.011 | 0.491 | 0.566 |
| WSR | ~~ | age | 0.855 | 0.859 | 0.068 | 0.655 | 0.998 | -0.02 | -0.02 | 0.015 | -0.05 | 0.053 |
| height | ~~ | weight | 0 | 0 | 0 | 0 | 0 | 0.528 | 0.528 | 0.013 | 0.49 | 0.593 |
| height | ~~ | age | 0.551 | 0.541 | 0.066 | 0.348 | 0.701 | -0.07 | -0.07 | 0.012 | -0.11 | -0.05 |
| weight | ~~ | age | 0.983 | 0.945 | 0.057 | 0.745 | 0.999 | 0.001 | 0.001 | 0.012 | -0.03 | 0.039 |
| sing F0 | ~ | WSR | 0.481 | 0.483 | 0.091 | 0.094 | 0.759 | 0.087 | 0.087 | 0.021 | 0.035 | 0.204 |
| sing F0 | ~ | height | 0.3 | 0.318 | 0.078 | 0.138 | 0.602 | -0.12 | -0.12 | 0.02 | -0.18 | -0.06 |
| sing F0 | ~ | weight | 0.902 | 0.858 | 0.094 | 0.466 | 0.999 | 0.016 | 0.017 | 0.021 | -0.08 | 0.09 |
| sing F0 | ~ | age | 0.533 | 0.538 | 0.075 | 0.316 | 0.788 | 0.075 | 0.075 | 0.014 | 0.031 | 0.121 |
| sing range | ~ | WSR | 0.419 | 0.429 | 0.087 | 0.263 | 0.791 | -0.1 | -0.1 | 0.017 | -0.14 | -0.03 |
| sing range | ~ | height | 0.84 | 0.842 | 0.095 | 0.475 | 0.999 | 0.023 | 0.023 | 0.018 | -0.04 | 0.088 |
| sing range | ~ | weight | 0.429 | 0.446 | 0.093 | 0.231 | 0.831 | 0.094 | 0.094 | 0.018 | 0.025 | 0.144 |
| sing range | ~ | age | 0.813 | 0.812 | 0.057 | 0.642 | 0.976 | -0.03 | -0.03 | 0.013 | -0.06 | 0.037 |
| talk F0 | ~ | WSR | 0.56 | 0.577 | 0.103 | 0.098 | 0.803 | 0.069 | 0.069 | 0.022 | 0.029 | 0.2 |
| talk F0 | ~ | height | 0.105 | 0.11 | 0.032 | 0.041 | 0.262 | -0.2 | -0.2 | 0.017 | -0.24 | -0.13 |
| talk F0 | ~ | weight | 0.941 | 0.906 | 0.101 | 0.371 | 1 | -0.01 | -0.01 | 0.021 | -0.11 | 0.037 |
| talk F0 | ~ | age | 0.791 | 0.788 | 0.084 | 0.426 | 0.96 | 0.031 | 0.031 | 0.016 | -0.04 | 0.097 |
| talk range | ~ | WSR | 0.593 | 0.582 | 0.099 | 0.207 | 0.871 | -0.07 | -0.07 | 0.021 | -0.15 | 0.02 |
| talk range | ~ | height | 0.363 | 0.371 | 0.09 | 0.198 | 0.758 | 0.11 | 0.111 | 0.02 | 0.038 | 0.158 |
| talk range | ~ | weight | 0.403 | 0.4 | 0.093 | 0.147 | 0.703 | -0.1 | -0.11 | 0.021 | -0.17 | -0.05 |
| talk range | ~ | age | 0.653 | 0.664 | 0.081 | 0.478 | 0.952 | 0.054 | 0.054 | 0.014 | 0.007 | 0.088 |
| sing ATTR | ~ | sing F0 | 0.713 | 0.711 | 0.088 | 0.542 | 0.976 | -0.05 | -0.05 | 0.015 | -0.08 | -0 |
| sing ATTR | ~ | sing range | 0.068 | 0.074 | 0.022 | 0.029 | 0.177 | 0.221 | 0.221 | 0.015 | 0.169 | 0.267 |
| sing ATTR | ~ | WSR | 0.209 | 0.211 | 0.052 | 0.086 | 0.386 | -0.16 | -0.16 | 0.017 | -0.2 | -0.11 |
| sing ATTR | ~ | height | 0.238 | 0.248 | 0.061 | 0.134 | 0.487 | -0.14 | -0.14 | 0.017 | -0.19 | -0.09 |
| sing ATTR | ~ | weight | 0 | 0 | 0 | 0 | 0.001 | 0.434 | 0.434 | 0.017 | 0.402 | 0.502 |
| sing ATTR | ~ | age | 0.799 | 0.793 | 0.087 | 0.33 | 0.964 | -0.03 | -0.03 | 0.016 | -0.12 | 0.015 |
| talk ATTR | ~ | talk F0 | 0.001 | 0.003 | 0.001 | 0.001 | 0.007 | -0.36 | -0.36 | 0.012 | -0.41 | -0.32 |
| talk ATTR | ~ | talk range | 0.025 | 0.026 | 0.007 | 0.004 | 0.046 | 0.264 | 0.265 | 0.012 | 0.241 | 0.332 |
| talk ATTR | ~ | WSR | 0.369 | 0.389 | 0.061 | 0.198 | 0.582 | -0.1 | -0.1 | 0.014 | -0.15 | -0.06 |
| talk ATTR | ~ | height | 0.833 | 0.823 | 0.081 | 0.539 | 0.998 | -0.03 | -0.03 | 0.016 | -0.07 | 0.034 |
| talk ATTR | ~ | weight | 0 | 0.001 | 0.001 | 0 | 0.003 | 0.4 | 0.4 | 0.016 | 0.348 | 0.442 |
| talk ATTR | ~ | age | 0.028 | 0.028 | 0.008 | 0.013 | 0.063 | -0.26 | -0.26 | 0.011 | -0.29 | -0.22 |
| SOI total | ~ | sing ATTR | 0.487 | 0.5 | 0.109 | 0.246 | 0.864 | -0.07 | -0.07 | 0.018 | -0.12 | -0.02 |
| SOI total | ~ | sing F0 | 0.002 | 0.003 | 0.003 | 0 | 0.027 | 0.311 | 0.311 | 0.019 | 0.238 | 0.39 |
| SOI total | ~ | sing range | 0.428 | 0.439 | 0.089 | 0.211 | 0.901 | -0.08 | -0.08 | 0.015 | -0.12 | -0.02 |
| SOI total | ~ | talk ATTR | 0.033 | 0.035 | 0.018 | 0.006 | 0.128 | 0.231 | 0.231 | 0.02 | 0.167 | 0.283 |
| SOI total | ~ | talk F0 | 0 | 0 | 0 | 0 | 0.001 | -0.42 | -0.42 | 0.017 | -0.49 | -0.38 |
| SOI total | ~ | talk range | 0.115 | 0.118 | 0.028 | 0.031 | 0.196 | 0.166 | 0.166 | 0.013 | 0.137 | 0.221 |
| SOI total | ~ | WSR | 0.152 | 0.162 | 0.047 | 0.022 | 0.352 | -0.15 | -0.15 | 0.017 | -0.24 | -0.1 |
| SOI total | ~ | height | 0.072 | 0.079 | 0.022 | 0.009 | 0.138 | -0.19 | -0.19 | 0.016 | -0.27 | -0.16 |
| SOI total | ~ | weight | 0.113 | 0.119 | 0.038 | 0.012 | 0.319 | 0.166 | 0.166 | 0.019 | 0.105 | 0.253 |
| SOI total | ~ | age | 0.001 | 0.002 | 0.001 | 0 | 0.006 | 0.322 | 0.322 | 0.014 | 0.268 | 0.353 |
|  |  |  |  |  |  |  |  |  |  |  |  |  |
| JK = jackknife mean estimation, SD = jackknife estimation SD, min. = jackknife minimum, max. = jackknife maximum,~~ indicates correlation, ~ regression coefficient, p (R)= robust p values from permutation test, std. = standardized parameter value, WSR=waist-to-shoulder ratio, ATTR = attractiveness, F0 = average fundamental frequency | | | | | | | | | | | | |

| **Table S9.** Jackknife estimations of robust p values and standardized coefficients in female sample | | | | | | | | | | | | |
| --- | --- | --- | --- | --- | --- | --- | --- | --- | --- | --- | --- | --- |
| Relationship |  |  | p (R) | JK | SD | min. | max. | std. | JK | SD | min. | max. |
| WHR | ~~ | height | 0.128 | 0.138 | 0.044 | 0.111 | 0.521 | 0.21 | 0.208 | 0.032 | -0.08 | 0.222 |
| WHR | ~~ | weight | 0.84 | 0.824 | 0.094 | 0.012 | 0.871 | -0 | 0.002 | 0.032 | -0 | 0.285 |
| WHR | ~~ | age | 0.291 | 0.286 | 0.026 | 0.25 | 0.492 | 0.158 | 0.156 | 0.027 | -0.08 | 0.171 |
| height | ~~ | weight | 0 | 0 | 0 | 0 | 0 | 0.474 | 0.474 | 0.013 | 0.407 | 0.502 |
| height | ~~ | age | 0.903 | 0.885 | 0.067 | 0.641 | 0.994 | -0.01 | -0.01 | 0.014 | -0.06 | 0.048 |
| weight | ~~ | age | 0.661 | 0.656 | 0.078 | 0.38 | 1 | 0.048 | 0.048 | 0.015 | -0.02 | 0.098 |
| sing F0 | ~ | WHR | 0.434 | 0.434 | 0.043 | 0.387 | 0.784 | 0.074 | 0.074 | 0.006 | 0.032 | 0.084 |
| sing F0 | ~ | height | 0.656 | 0.665 | 0.085 | 0.468 | 0.996 | -0.05 | -0.05 | 0.014 | -0.08 | 0.001 |
| sing F0 | ~ | weight | 0.052 | 0.048 | 0.011 | 0.021 | 0.082 | -0.23 | -0.23 | 0.011 | -0.27 | -0.2 |
| sing F0 | ~ | age | 0.862 | 0.86 | 0.067 | 0.444 | 0.985 | -0.02 | -0.02 | 0.013 | -0.09 | 0.022 |
| sing range | ~ | WHR | 0.976 | 0.961 | 0.075 | 0.339 | 1 | -0.02 | -0.02 | 0.015 | -0.04 | 0.109 |
| sing range | ~ | height | 0.161 | 0.169 | 0.044 | 0.055 | 0.332 | 0.162 | 0.162 | 0.016 | 0.114 | 0.225 |
| sing range | ~ | weight | 0.053 | 0.055 | 0.017 | 0.009 | 0.117 | -0.22 | -0.22 | 0.016 | -0.29 | -0.18 |
| sing range | ~ | age | 0.368 | 0.379 | 0.059 | 0.195 | 0.548 | -0.1 | -0.1 | 0.012 | -0.15 | -0.07 |
| talk F0 | ~ | WHR | 0.862 | 0.857 | 0.024 | 0.805 | 0.949 | -0.01 | -0.01 | 0.004 | -0.02 | 0.009 |
| talk F0 | ~ | height | 0.187 | 0.198 | 0.044 | 0.094 | 0.366 | -0.15 | -0.15 | 0.014 | -0.2 | -0.11 |
| talk F0 | ~ | weight | 0.431 | 0.436 | 0.068 | 0.266 | 0.736 | -0.09 | -0.09 | 0.014 | -0.14 | -0.04 |
| talk F0 | ~ | age | 0.187 | 0.192 | 0.035 | 0.071 | 0.281 | -0.15 | -0.15 | 0.013 | -0.21 | -0.13 |
| talk range | ~ | WHR | 0.225 | 0.21 | 0.025 | 0.069 | 0.257 | -0.16 | -0.15 | 0.042 | -0.17 | 0.214 |
| talk range | ~ | height | 0.082 | 0.089 | 0.029 | 0.022 | 0.24 | -0.2 | -0.2 | 0.016 | -0.26 | -0.14 |
| talk range | ~ | weight | 0.506 | 0.514 | 0.093 | 0.25 | 0.954 | 0.077 | 0.076 | 0.019 | -0.01 | 0.134 |
| talk range | ~ | age | 0.948 | 0.922 | 0.07 | 0.63 | 1 | 0.008 | 0.008 | 0.013 | -0.01 | 0.055 |
| sing ATTR | ~ | sing F0 | 0 | 0 | 0 | 0 | 0 | 0.516 | 0.516 | 0.01 | 0.488 | 0.547 |
| sing ATTR | ~ | sing range | 0.166 | 0.17 | 0.032 | 0.09 | 0.279 | -0.16 | -0.16 | 0.012 | -0.2 | -0.13 |
| sing ATTR | ~ | WHR | 0.835 | 0.838 | 0.035 | 0.728 | 0.966 | 0.024 | 0.024 | 0.004 | 0.007 | 0.035 |
| sing ATTR | ~ | height | 0.738 | 0.732 | 0.085 | 0.462 | 0.958 | -0.04 | -0.04 | 0.013 | -0.09 | -0.01 |
| sing ATTR | ~ | weight | 0.187 | 0.184 | 0.043 | 0.115 | 0.477 | -0.16 | -0.16 | 0.013 | -0.19 | -0.08 |
| sing ATTR | ~ | age | 0.694 | 0.699 | 0.064 | 0.567 | 0.974 | -0.05 | -0.05 | 0.011 | -0.07 | 0.003 |
| talk ATTR | ~ | talk F0 | 0.001 | 0.001 | 0 | 0 | 0.003 | 0.403 | 0.403 | 0.013 | 0.366 | 0.461 |
| talk ATTR | ~ | talk range | 0.749 | 0.742 | 0.078 | 0.434 | 0.961 | 0.039 | 0.039 | 0.013 | -0.01 | 0.095 |
| talk ATTR | ~ | WHR | 0.207 | 0.219 | 0.076 | 0.17 | 0.869 | 0.138 | 0.137 | 0.018 | -0.02 | 0.151 |
| talk ATTR | ~ | height | 0.43 | 0.433 | 0.072 | 0.262 | 0.686 | -0.09 | -0.09 | 0.014 | -0.13 | -0.05 |
| talk ATTR | ~ | weight | 0.232 | 0.241 | 0.039 | 0.102 | 0.388 | -0.14 | -0.14 | 0.012 | -0.2 | -0.1 |
| talk ATTR | ~ | age | 0.81 | 0.788 | 0.063 | 0.6 | 0.938 | -0.03 | -0.03 | 0.011 | -0.06 | 0.011 |
| SOI total | ~ | sing ATTR | 0.193 | 0.204 | 0.073 | 0.056 | 0.458 | -0.16 | -0.16 | 0.024 | -0.23 | -0.09 |
| SOI total | ~ | sing F0 | 0.332 | 0.341 | 0.074 | 0.152 | 0.572 | 0.116 | 0.116 | 0.018 | 0.068 | 0.17 |
| SOI total | ~ | sing range | 0.081 | 0.083 | 0.03 | 0.038 | 0.242 | 0.212 | 0.212 | 0.017 | 0.138 | 0.253 |
| SOI total | ~ | talk ATTR | 0.177 | 0.185 | 0.059 | 0.071 | 0.466 | 0.162 | 0.162 | 0.019 | 0.09 | 0.213 |
| SOI total | ~ | talk F0 | 0.13 | 0.136 | 0.038 | 0.053 | 0.266 | -0.18 | -0.18 | 0.017 | -0.23 | -0.14 |
| SOI total | ~ | talk range | 0.382 | 0.383 | 0.059 | 0.182 | 0.571 | 0.106 | 0.106 | 0.013 | 0.068 | 0.159 |
| SOI total | ~ | WHR | 0.804 | 0.786 | 0.063 | 0.54 | 0.989 | 0.024 | 0.023 | 0.014 | -0.08 | 0.05 |
| SOI total | ~ | height | 0.223 | 0.233 | 0.056 | 0.063 | 0.39 | 0.147 | 0.147 | 0.02 | 0.103 | 0.229 |
| SOI total | ~ | weight | 0.895 | 0.88 | 0.088 | 0.496 | 0.999 | -0.02 | -0.02 | 0.016 | -0.08 | 0.015 |
| SOI total | ~ | age | 0.826 | 0.833 | 0.077 | 0.496 | 0.982 | 0.023 | 0.023 | 0.015 | -0.03 | 0.081 |
|  |  |  |  |  |  |  |  |  |  |  |  |  |
| JK = jackknife mean estimation, SD = jackknife estimation SD, min. = jackknife minimum, max. = jackknife maximum,~~ indicates correlation, ~ regression coefficient, p (R)= robust p values from permutation test, std. = standardized parameter value, WHR=waist-to-hip ratio, ATTR = attractiveness, F0 = average fundamental frequency | | | | | | | | | | | | |
|  |  |  |  |  |  |  |  |  |  |  |  |  |

**Full model estimations with VTL**

| **Table S10.** Estimation of the full structural model for male participants | | | | | | | | | | |
| --- | --- | --- | --- | --- | --- | --- | --- | --- | --- | --- |
| Relationship |  |  | est. | SE | z | p (A) | p (R) | CI lower | CI upper | std. |
| WSR | ~~ | height | 0.01 | 0.042 | 0.23 | 0.818 | 0.816 | -0.072 | 0.091 | 0.027 |
| WSR | ~~ | weight | 0.292 | 0.073 | 3.977 | 0 | 0 | 0.148 | 0.435 | 0.526 |
| WSR | ~~ | age | -0.003 | 0.019 | -0.171 | 0.864 | 0.87 | -0.041 | 0.034 | -0.02 |
| height | ~~ | weight | 39.111 | 9.803 | 3.99 | 0 | 0 | 19.898 | 58.324 | 0.528 |
| height | ~~ | age | -1.607 | 2.561 | -0.627 | 0.53 | 0.524 | -6.627 | 3.413 | -0.074 |
| weight | ~~ | age | 0.045 | 3.976 | 0.011 | 0.991 | 0.993 | -7.747 | 7.837 | 0.001 |
| sing VTL | ~ | WSR | -0.761 | 1.611 | -0.472 | 0.637 | 0.588 | -3.919 | 2.397 | -0.066 |
| sing VTL | ~ | height | 0.004 | 0.012 | 0.311 | 0.756 | 0.721 | -0.02 | 0.028 | 0.044 |
| sing VTL | ~ | weight | 0.017 | 0.009 | 1.879 | 0.06 | 0.008 | -0.001 | 0.035 | 0.31 |
| sing VTL | ~ | age | 0.001 | 0.021 | 0.051 | 0.959 | 0.966 | -0.04 | 0.042 | 0.006 |
| sing range | ~ | WSR | -4.624 | 6.925 | -0.668 | 0.504 | 0.423 | -18.196 | 8.949 | -0.098 |
| sing range | ~ | height | 0.008 | 0.052 | 0.154 | 0.878 | 0.843 | -0.094 | 0.11 | 0.023 |
| sing range | ~ | weight | 0.021 | 0.039 | 0.546 | 0.585 | 0.431 | -0.056 | 0.098 | 0.094 |
| sing range | ~ | age | -0.021 | 0.091 | -0.237 | 0.813 | 0.828 | -0.199 | 0.156 | -0.028 |
| talk VTL | ~ | WSR | -0.888 | 1.282 | -0.693 | 0.489 | 0.48 | -3.4 | 1.624 | -0.09 |
| talk VTL | ~ | height | 0.016 | 0.01 | 1.668 | 0.095 | 0.068 | -0.003 | 0.035 | 0.217 |
| talk VTL | ~ | weight | 0.016 | 0.007 | 2.236 | 0.025 | 0.003 | 0.002 | 0.03 | 0.341 |
| talk VTL | ~ | age | 0.017 | 0.017 | 0.999 | 0.318 | 0.385 | -0.016 | 0.05 | 0.104 |
| talk range | ~ | WSR | -3.541 | 7.601 | -0.466 | 0.641 | 0.585 | -18.438 | 11.356 | -0.068 |
| talk range | ~ | height | 0.043 | 0.057 | 0.757 | 0.449 | 0.363 | -0.069 | 0.155 | 0.11 |
| talk range | ~ | weight | -0.026 | 0.043 | -0.611 | 0.541 | 0.386 | -0.111 | 0.058 | -0.104 |
| talk range | ~ | age | 0.046 | 0.099 | 0.463 | 0.643 | 0.659 | -0.149 | 0.241 | 0.054 |
| sing ATTR | ~ | sing VTL | -0.067 | 0.136 | -0.492 | 0.623 | 0.646 | -0.333 | 0.199 | -0.056 |
| sing ATTR | ~ | sing range | 0.054 | 0.032 | 1.705 | 0.088 | 0.123 | -0.008 | 0.116 | 0.185 |
| sing ATTR | ~ | WSR | -2.317 | 1.879 | -1.233 | 0.218 | 0.171 | -6 | 1.366 | -0.168 |
| sing ATTR | ~ | height | -0.014 | 0.014 | -0.994 | 0.32 | 0.27 | -0.042 | 0.014 | -0.136 |
| sing ATTR | ~ | weight | 0.03 | 0.011 | 2.783 | 0.005 | 0 | 0.009 | 0.052 | 0.457 |
| sing ATTR | ~ | age | -0.008 | 0.024 | -0.337 | 0.736 | 0.782 | -0.056 | 0.04 | -0.037 |
| talk ATTR | ~ | talk VTL | 0.187 | 0.149 | 1.26 | 0.208 | 0.245 | -0.104 | 0.478 | 0.143 |
| talk ATTR | ~ | talk range | 0.047 | 0.025 | 1.86 | 0.063 | 0.12 | -0.003 | 0.096 | 0.189 |
| talk ATTR | ~ | WSR | -1.608 | 1.634 | -0.984 | 0.325 | 0.311 | -4.812 | 1.595 | -0.124 |
| talk ATTR | ~ | height | 0.002 | 0.013 | 0.182 | 0.855 | 0.851 | -0.022 | 0.027 | 0.024 |
| talk ATTR | ~ | weight | 0.022 | 0.01 | 2.316 | 0.021 | 0.005 | 0.003 | 0.041 | 0.356 |
| talk ATTR | ~ | age | -0.061 | 0.021 | -2.865 | 0.004 | 0.016 | -0.103 | -0.019 | -0.291 |
| SOI total | ~ | sing ATTR | -1.057 | 2.299 | -0.46 | 0.646 | 0.705 | -5.562 | 3.448 | -0.048 |
| SOI total | ~ | sing VTL | -0.993 | 2.673 | -0.372 | 0.71 | 0.751 | -6.232 | 4.245 | -0.038 |
| SOI total | ~ | sing range | -0.377 | 0.633 | -0.596 | 0.551 | 0.65 | -1.618 | 0.864 | -0.059 |
| SOI total | ~ | talk ATTR | 9.672 | 2.643 | 3.659 | 0 | 0.001 | 4.491 | 14.853 | 0.416 |
| SOI total | ~ | talk VTL | -4.461 | 3.391 | -1.315 | 0.188 | 0.248 | -11.108 | 2.186 | -0.147 |
| SOI total | ~ | talk range | 0.115 | 0.579 | 0.199 | 0.842 | 0.869 | -1.02 | 1.25 | 0.02 |
| SOI total | ~ | WSR | -53.15 | 37.7 | -1.41 | 0.159 | 0.155 | -127.046 | 20.737 | -0.177 |
| SOI total | ~ | height | -0.279 | 0.285 | -0.978 | 0.328 | 0.322 | -0.837 | 0.28 | -0.124 |
| SOI total | ~ | weight | 0.254 | 0.239 | 1.062 | 0.288 | 0.168 | -0.214 | 0.722 | 0.175 |
| SOI total | ~ | age | 2.281 | 0.511 | 4.462 | 0 | 0 | 1.279 | 3.282 | 0.464 |
|  |  |  |  |  |  |  |  |  |  |  |
| ~~ indicates correlation, ~ regression coefficient, est.= estimated parameter, SE = standard error, p (A) = probability of the relationship being insignificant (standard lavaan output), p (R)= robust p value from permutation test, CI = 95% confidence interval of estimated parameter, std. = standardized parameter value, WSR=waist-to-shoulder ratio, ATTR = attractiveness, VTL = apparent vocal tract length   | **Table S11.** Estimation of the full structural model for female participants | | | | | | | | | | | | --- | --- | --- | --- | --- | --- | --- | --- | --- | --- | --- | | Relationship |  |  | est. | SE | z | p (A) | p (R) | CI lower | CI upper | std. | | WHR | ~~ | height | 13.749 | 7.52 | 1.828 | 0.067 | 0.136 | -0.989 | 28.488 | 0.21 | | WHR | ~~ | weight | -0.197 | 13.571 | -0.015 | 0.988 | 0.834 | -26.796 | 26.401 | -0.002 | | WHR | ~~ | age | 7.168 | 5.157 | 1.39 | 0.165 | 0.278 | -2.94 | 17.275 | 0.158 | | height | ~~ | weight | 31.023 | 8.142 | 3.81 | 0 | 0 | 15.065 | 46.981 | 0.475 | | height | ~~ | age | -0.345 | 2.761 | -0.125 | 0.901 | 0.898 | -5.757 | 5.067 | -0.014 | | weight | ~~ | age | 2.175 | 5.098 | 0.427 | 0.67 | 0.661 | -7.816 | 12.166 | 0.048 | | sing VTL | ~ | WHR | 0 | 0.004 | 0.127 | 0.899 | 0.938 | -0.007 | 0.008 | 0.015 | | sing VTL | ~ | height | 0.008 | 0.008 | 1 | 0.317 | 0.27 | -0.008 | 0.023 | 0.13 | | sing VTL | ~ | weight | 0.003 | 0.004 | 0.727 | 0.467 | 0.442 | -0.005 | 0.011 | 0.092 | | sing VTL | ~ | age | -0.003 | 0.01 | -0.277 | 0.782 | 0.789 | -0.022 | 0.016 | -0.031 | | sing range | ~ | WHR | -0.006 | 0.032 | -0.177 | 0.859 | 0.954 | -0.069 | 0.057 | -0.02 | | sing range | ~ | height | 0.084 | 0.067 | 1.257 | 0.209 | 0.168 | -0.047 | 0.214 | 0.162 | | sing range | ~ | weight | -0.061 | 0.035 | -1.734 | 0.083 | 0.052 | -0.13 | 0.008 | -0.218 | | sing range | ~ | age | -0.078 | 0.083 | -0.935 | 0.35 | 0.368 | -0.241 | 0.085 | -0.104 | | talk VTL | ~ | WHR | -0.002 | 0.004 | -0.584 | 0.559 | 0.546 | -0.009 | 0.005 | -0.067 | | talk VTL | ~ | height | 0.013 | 0.008 | 1.703 | 0.088 | 0.056 | -0.002 | 0.028 | 0.22 | | talk VTL | ~ | weight | -0.004 | 0.004 | -0.89 | 0.373 | 0.333 | -0.012 | 0.004 | -0.112 | | talk VTL | ~ | age | -0.006 | 0.01 | -0.624 | 0.532 | 0.533 | -0.025 | 0.013 | -0.07 | | talk range | ~ | WHR | -0.056 | 0.041 | -1.368 | 0.171 | 0.21 | -0.136 | 0.024 | -0.155 | | talk range | ~ | height | -0.133 | 0.085 | -1.569 | 0.117 | 0.09 | -0.298 | 0.033 | -0.2 | | talk range | ~ | weight | 0.028 | 0.045 | 0.62 | 0.536 | 0.515 | -0.06 | 0.116 | 0.077 | | talk range | ~ | age | 0.007 | 0.106 | 0.07 | 0.945 | 0.934 | -0.2 | 0.215 | 0.008 | | sing ATTR | ~ | sing VTL | -0.304 | 0.199 | -1.532 | 0.125 | 0.169 | -0.693 | 0.085 | -0.162 | | sing ATTR | ~ | sing range | -0.042 | 0.023 | -1.817 | 0.069 | 0.094 | -0.088 | 0.003 | -0.194 | | sing ATTR | ~ | WHR | 0.004 | 0.007 | 0.585 | 0.559 | 0.539 | -0.009 | 0.017 | 0.063 | | sing ATTR | ~ | height | -0.005 | 0.014 | -0.332 | 0.74 | 0.72 | -0.032 | 0.023 | -0.041 | | sing ATTR | ~ | weight | -0.016 | 0.007 | -2.19 | 0.029 | 0.019 | -0.031 | -0.002 | -0.266 | | sing ATTR | ~ | age | -0.011 | 0.017 | -0.608 | 0.543 | 0.59 | -0.045 | 0.023 | -0.065 | | talk ATTR | ~ | talk VTL | -0.4 | 0.19 | -2.107 | 0.035 | 0.059 | -0.771 | -0.028 | -0.224 | | talk ATTR | ~ | talk range | 0.008 | 0.017 | 0.491 | 0.624 | 0.67 | -0.025 | 0.042 | 0.053 | | talk ATTR | ~ | WHR | 0.007 | 0.006 | 1.097 | 0.273 | 0.281 | -0.005 | 0.019 | 0.121 | | talk ATTR | ~ | height | -0.011 | 0.013 | -0.817 | 0.414 | 0.367 | -0.037 | 0.015 | -0.103 | | talk ATTR | ~ | weight | -0.012 | 0.007 | -1.699 | 0.089 | 0.09 | -0.025 | 0.002 | -0.204 | | talk ATTR | ~ | age | -0.017 | 0.016 | -1.024 | 0.306 | 0.355 | -0.048 | 0.015 | -0.108 | | SOI total | ~ | sing ATTR | -1.519 | 2.065 | -0.736 | 0.462 | 0.517 | -5.566 | 2.528 | -0.071 | | SOI total | ~ | sing VTL | 11.377 | 3.697 | 3.077 | 0.002 | 0.009 | 4.13 | 18.623 | 0.282 | | SOI total | ~ | sing range | 1.062 | 0.437 | 2.43 | 0.015 | 0.043 | 0.205 | 1.918 | 0.226 | | SOI total | ~ | talk ATTR | -1.243 | 2.211 | -0.562 | 0.574 | 0.617 | -5.575 | 3.09 | -0.054 | | SOI total | ~ | talk VTL | -19.48 | 3.831 | -5.086 | 0 | 0 | -26.992 | -11.976 | -0.474 | | SOI total | ~ | talk range | 0.377 | 0.337 | 1.118 | 0.263 | 0.358 | -0.284 | 1.039 | 0.103 | | SOI total | ~ | WHR | 0.024 | 0.125 | 0.193 | 0.847 | 0.786 | -0.221 | 0.269 | 0.018 | | SOI total | ~ | height | 0.467 | 0.267 | 1.751 | 0.08 | 0.079 | -0.056 | 0.99 | 0.192 | | SOI total | ~ | weight | -0.155 | 0.145 | -1.069 | 0.285 | 0.281 | -0.438 | 0.129 | -0.117 | | SOI total | ~ | age | 0.028 | 0.322 | 0.085 | 0.932 | 0.947 | -0.604 | 0.66 | 0.008 | | ~~ indicates correlation, ~ regression coefficient, est.= estimated parameter, SE = standard error, p (A) = probability of the relationship being insignificant (standard lavaan output), p (R)= robust p value from permutation test, CI = 95% confidence interval of estimated parameter, std. = standardized parameter value, WHR=waist-to-hip ratio, ATTR = attractiveness, VTL = apparent vocal tract length | | | | | | | | | | | | | | | | | | | | | |

**Jackknife estimates and ranges of robust p values and standardized coefficients yielded by leaving out one observation at a time**

| **Table S12.** Jackknife estimations of robust p values and standardized coefficients in male sample | | | | | | | | | | | | |
| --- | --- | --- | --- | --- | --- | --- | --- | --- | --- | --- | --- | --- |
| Relationship |  |  | p (R) | JK | SD | min. | max. | std. | JK | SD | min. | max. |
| WSR | ~~ | height | 0.816 | 0.815 | 0.083 | 0.526 | 0.991 | 0.027 | 0.027 | 0.015 | -0.02 | 0.076 |
| WSR | ~~ | weight | 0 | 0 | 0 | 0 | 0 | 0.526 | 0.526 | 0.011 | 0.491 | 0.566 |
| WSR | ~~ | age | 0.87 | 0.86 | 0.07 | 0.665 | 0.994 | -0.02 | -0.02 | 0.015 | -0.05 | 0.053 |
| height | ~~ | weight | 0 | 0 | 0 | 0 | 0 | 0.528 | 0.528 | 0.013 | 0.49 | 0.593 |
| height | ~~ | age | 0.524 | 0.542 | 0.064 | 0.335 | 0.688 | -0.07 | -0.07 | 0.012 | -0.11 | -0.05 |
| weight | ~~ | age | 0.993 | 0.946 | 0.06 | 0.746 | 0.999 | 0.001 | 0.001 | 0.012 | -0.03 | 0.039 |
| sing VTL | ~ | WSR | 0.588 | 0.593 | 0.084 | 0.408 | 0.882 | -0.07 | -0.07 | 0.015 | -0.1 | -0.02 |
| sing VTL | ~ | height | 0.721 | 0.723 | 0.106 | 0.285 | 0.953 | 0.044 | 0.044 | 0.019 | -0.01 | 0.13 |
| sing VTL | ~ | weight | 0.008 | 0.012 | 0.01 | 0.001 | 0.067 | 0.31 | 0.309 | 0.022 | 0.225 | 0.368 |
| sing VTL | ~ | age | 0.966 | 0.93 | 0.074 | 0.545 | 0.999 | 0.006 | 0.006 | 0.015 | -0.06 | 0.076 |
| sing range | ~ | WSR | 0.423 | 0.431 | 0.086 | 0.263 | 0.795 | -0.1 | -0.1 | 0.017 | -0.14 | -0.03 |
| sing range | ~ | height | 0.843 | 0.844 | 0.095 | 0.469 | 0.992 | 0.023 | 0.023 | 0.018 | -0.04 | 0.088 |
| sing range | ~ | weight | 0.431 | 0.446 | 0.095 | 0.236 | 0.848 | 0.094 | 0.094 | 0.018 | 0.025 | 0.145 |
| sing range | ~ | age | 0.828 | 0.812 | 0.058 | 0.644 | 0.982 | -0.03 | -0.03 | 0.013 | -0.06 | 0.037 |
| talk VTL | ~ | WSR | 0.48 | 0.468 | 0.077 | 0.295 | 0.714 | -0.09 | -0.09 | 0.015 | -0.13 | -0.05 |
| talk VTL | ~ | height | 0.068 | 0.077 | 0.025 | 0.024 | 0.181 | 0.217 | 0.217 | 0.017 | 0.166 | 0.275 |
| talk VTL | ~ | weight | 0.003 | 0.005 | 0.003 | 0.001 | 0.022 | 0.341 | 0.341 | 0.021 | 0.28 | 0.412 |
| talk VTL | ~ | age | 0.385 | 0.399 | 0.07 | 0.211 | 0.667 | 0.104 | 0.104 | 0.015 | 0.049 | 0.152 |
| talk range | ~ | WSR | 0.585 | 0.581 | 0.097 | 0.225 | 0.865 | -0.07 | -0.07 | 0.021 | -0.15 | 0.02 |
| talk range | ~ | height | 0.363 | 0.371 | 0.091 | 0.201 | 0.756 | 0.11 | 0.11 | 0.02 | 0.037 | 0.158 |
| talk range | ~ | weight | 0.386 | 0.398 | 0.092 | 0.156 | 0.69 | -0.1 | -0.1 | 0.021 | -0.17 | -0.05 |
| talk range | ~ | age | 0.659 | 0.663 | 0.082 | 0.459 | 0.949 | 0.054 | 0.054 | 0.014 | 0.007 | 0.088 |
| sing ATTR | ~ | sing VTL | 0.646 | 0.658 | 0.085 | 0.447 | 0.971 | -0.06 | -0.06 | 0.015 | -0.1 | -0.01 |
| sing ATTR | ~ | sing range | 0.123 | 0.136 | 0.031 | 0.056 | 0.281 | 0.185 | 0.186 | 0.014 | 0.134 | 0.235 |
| sing ATTR | ~ | WSR | 0.171 | 0.179 | 0.048 | 0.077 | 0.349 | -0.17 | -0.17 | 0.018 | -0.22 | -0.12 |
| sing ATTR | ~ | height | 0.27 | 0.278 | 0.062 | 0.147 | 0.489 | -0.14 | -0.14 | 0.016 | -0.18 | -0.09 |
| sing ATTR | ~ | weight | 0 | 0 | 0 | 0 | 0.001 | 0.457 | 0.457 | 0.019 | 0.41 | 0.538 |
| sing ATTR | ~ | age | 0.782 | 0.769 | 0.089 | 0.291 | 0.958 | -0.04 | -0.04 | 0.017 | -0.13 | 0.01 |
| talk ATTR | ~ | talk VTL | 0.245 | 0.247 | 0.049 | 0.132 | 0.415 | 0.143 | 0.143 | 0.014 | 0.101 | 0.181 |
| talk ATTR | ~ | talk range | 0.12 | 0.124 | 0.023 | 0.045 | 0.192 | 0.189 | 0.189 | 0.012 | 0.16 | 0.242 |
| talk ATTR | ~ | WSR | 0.311 | 0.316 | 0.063 | 0.146 | 0.509 | -0.12 | -0.12 | 0.017 | -0.18 | -0.08 |
| talk ATTR | ~ | height | 0.851 | 0.842 | 0.091 | 0.48 | 0.994 | 0.024 | 0.024 | 0.017 | -0.01 | 0.088 |
| talk ATTR | ~ | weight | 0.005 | 0.003 | 0.002 | 0.001 | 0.016 | 0.356 | 0.356 | 0.019 | 0.293 | 0.409 |
| talk ATTR | ~ | age | 0.016 | 0.018 | 0.007 | 0.008 | 0.059 | -0.29 | -0.29 | 0.012 | -0.32 | -0.23 |
| SOI total | ~ | sing ATTR | 0.705 | 0.701 | 0.12 | 0.371 | 0.964 | -0.05 | -0.05 | 0.023 | -0.11 | 0.02 |
| SOI total | ~ | sing VTL | 0.751 | 0.756 | 0.093 | 0.465 | 0.935 | -0.04 | -0.04 | 0.021 | -0.1 | 0.058 |
| SOI total | ~ | sing range | 0.65 | 0.64 | 0.079 | 0.409 | 0.881 | -0.06 | -0.06 | 0.016 | -0.11 | 0.019 |
| SOI total | ~ | talk ATTR | 0.001 | 0.001 | 0.001 | 0 | 0.007 | 0.416 | 0.415 | 0.021 | 0.34 | 0.486 |
| SOI total | ~ | talk VTL | 0.248 | 0.254 | 0.075 | 0.084 | 0.565 | -0.15 | -0.15 | 0.023 | -0.21 | -0.07 |
| SOI total | ~ | talk range | 0.869 | 0.864 | 0.085 | 0.552 | 0.995 | 0.02 | 0.02 | 0.017 | -0.02 | 0.075 |
| SOI total | ~ | WSR | 0.155 | 0.17 | 0.052 | 0.025 | 0.396 | -0.18 | -0.18 | 0.021 | -0.28 | -0.11 |
| SOI total | ~ | height | 0.322 | 0.337 | 0.071 | 0.054 | 0.525 | -0.12 | -0.12 | 0.021 | -0.23 | -0.08 |
| SOI total | ~ | weight | 0.168 | 0.172 | 0.056 | 0.027 | 0.436 | 0.175 | 0.175 | 0.024 | 0.098 | 0.275 |
| SOI total | ~ | age | 0 | 0 | 0 | 0 | 0.001 | 0.464 | 0.464 | 0.011 | 0.437 | 0.489 |
|  |  |  |  |  |  |  |  |  |  |  |  |  |
| JK = jackknife mean estimation, SD = jackknife estimation SD, min. = jackknife minimum, max. = jackknife maximum,~~ indicates correlation, ~ regression coefficient, p (R)= robust p values from permutation test, std. = standardized parameter value, WSR=waist-to-shoulder ratio, ATTR = attractiveness, VTL = apparent vocal tract length | | | | | | | | | | | | |

| **Table S13.** Jackknife estimations of robust p values and standardized coefficients in female sample | | | | | | | | | | | | |
| --- | --- | --- | --- | --- | --- | --- | --- | --- | --- | --- | --- | --- |
| Relationship |  |  | p (R) | JK | SD | min. | max. | std. | JK | SD | min. | max. |
| WHR | ~~ | height | 0.136 | 0.135 | 0.044 | 0.104 | 0.513 | 0.21 | 0.208 | 0.032 | -0.08 | 0.221 |
| WHR | ~~ | weight | 0.834 | 0.826 | 0.094 | 0.014 | 0.874 | -0 | 0.002 | 0.032 | -0 | 0.285 |
| WHR | ~~ | age | 0.278 | 0.286 | 0.029 | 0.245 | 0.51 | 0.158 | 0.156 | 0.027 | -0.08 | 0.171 |
| height | ~~ | weight | 0 | 0 | 0 | 0 | 0 | 0.475 | 0.474 | 0.013 | 0.407 | 0.502 |
| height | ~~ | age | 0.898 | 0.882 | 0.067 | 0.626 | 0.997 | -0.01 | -0.01 | 0.014 | -0.06 | 0.048 |
| weight | ~~ | age | 0.661 | 0.655 | 0.076 | 0.4 | 0.986 | 0.048 | 0.048 | 0.015 | -0.02 | 0.098 |
| sing VTL | ~ | WHR | 0.938 | 0.933 | 0.065 | 0.43 | 0.996 | 0.015 | 0.013 | 0.013 | -0.09 | 0.028 |
| sing VTL | ~ | height | 0.27 | 0.273 | 0.056 | 0.148 | 0.48 | 0.13 | 0.129 | 0.014 | 0.084 | 0.169 |
| sing VTL | ~ | weight | 0.442 | 0.435 | 0.065 | 0.247 | 0.694 | 0.092 | 0.093 | 0.013 | 0.048 | 0.133 |
| sing VTL | ~ | age | 0.789 | 0.789 | 0.078 | 0.602 | 0.979 | -0.03 | -0.03 | 0.013 | -0.06 | 0.012 |
| sing range | ~ | WHR | 0.954 | 0.962 | 0.074 | 0.343 | 0.999 | -0.02 | -0.02 | 0.015 | -0.04 | 0.109 |
| sing range | ~ | height | 0.168 | 0.17 | 0.044 | 0.051 | 0.325 | 0.162 | 0.162 | 0.016 | 0.114 | 0.225 |
| sing range | ~ | weight | 0.052 | 0.056 | 0.018 | 0.009 | 0.128 | -0.22 | -0.22 | 0.016 | -0.29 | -0.18 |
| sing range | ~ | age | 0.368 | 0.378 | 0.057 | 0.209 | 0.546 | -0.1 | -0.1 | 0.012 | -0.15 | -0.07 |
| talk VTL | ~ | WHR | 0.546 | 0.537 | 0.02 | 0.484 | 0.618 | -0.07 | -0.07 | 0.016 | -0.08 | 0.069 |
| talk VTL | ~ | height | 0.056 | 0.061 | 0.022 | 0.031 | 0.192 | 0.22 | 0.22 | 0.015 | 0.152 | 0.253 |
| talk VTL | ~ | weight | 0.333 | 0.333 | 0.062 | 0.086 | 0.569 | -0.11 | -0.11 | 0.026 | -0.21 | 0.073 |
| talk VTL | ~ | age | 0.533 | 0.554 | 0.08 | 0.398 | 0.95 | -0.07 | -0.07 | 0.013 | -0.1 | -0.01 |
| talk range | ~ | WHR | 0.21 | 0.209 | 0.026 | 0.067 | 0.262 | -0.16 | -0.15 | 0.042 | -0.17 | 0.214 |
| talk range | ~ | height | 0.09 | 0.089 | 0.028 | 0.024 | 0.241 | -0.2 | -0.2 | 0.016 | -0.26 | -0.14 |
| talk range | ~ | weight | 0.515 | 0.516 | 0.093 | 0.263 | 0.954 | 0.077 | 0.076 | 0.019 | -0.01 | 0.134 |
| talk range | ~ | age | 0.934 | 0.921 | 0.07 | 0.636 | 0.999 | 0.008 | 0.008 | 0.013 | -0.01 | 0.055 |
| sing ATTR | ~ | sing VTL | 0.169 | 0.173 | 0.04 | 0.071 | 0.361 | -0.16 | -0.16 | 0.014 | -0.21 | -0.11 |
| sing ATTR | ~ | sing range | 0.094 | 0.101 | 0.024 | 0.033 | 0.168 | -0.19 | -0.19 | 0.013 | -0.25 | -0.16 |
| sing ATTR | ~ | WHR | 0.539 | 0.552 | 0.04 | 0.483 | 0.824 | 0.063 | 0.063 | 0.006 | 0.027 | 0.075 |
| sing ATTR | ~ | height | 0.72 | 0.732 | 0.102 | 0.411 | 1 | -0.04 | -0.04 | 0.016 | -0.1 | 0.002 |
| sing ATTR | ~ | weight | 0.019 | 0.022 | 0.008 | 0.009 | 0.079 | -0.27 | -0.27 | 0.013 | -0.3 | -0.2 |
| sing ATTR | ~ | age | 0.59 | 0.59 | 0.07 | 0.439 | 0.842 | -0.07 | -0.06 | 0.012 | -0.09 | -0.03 |
| talk ATTR | ~ | talk VTL | 0.059 | 0.061 | 0.03 | 0.016 | 0.293 | -0.22 | -0.22 | 0.017 | -0.28 | -0.13 |
| talk ATTR | ~ | talk range | 0.67 | 0.661 | 0.082 | 0.359 | 0.896 | 0.053 | 0.053 | 0.014 | 0.016 | 0.108 |
| talk ATTR | ~ | WHR | 0.281 | 0.288 | 0.075 | 0.211 | 0.925 | 0.121 | 0.12 | 0.015 | -0.01 | 0.133 |
| talk ATTR | ~ | height | 0.367 | 0.389 | 0.077 | 0.184 | 0.649 | -0.1 | -0.1 | 0.017 | -0.16 | -0.05 |
| talk ATTR | ~ | weight | 0.09 | 0.094 | 0.031 | 0.022 | 0.316 | -0.2 | -0.2 | 0.016 | -0.27 | -0.12 |
| talk ATTR | ~ | age | 0.355 | 0.361 | 0.047 | 0.217 | 0.508 | -0.11 | -0.11 | 0.011 | -0.15 | -0.08 |
| SOI total | ~ | sing ATTR | 0.517 | 0.53 | 0.093 | 0.318 | 0.865 | -0.07 | -0.07 | 0.015 | -0.11 | -0.02 |
| SOI total | ~ | sing VTL | 0.009 | 0.01 | 0.005 | 0.001 | 0.025 | 0.282 | 0.283 | 0.015 | 0.249 | 0.342 |
| SOI total | ~ | sing range | 0.043 | 0.044 | 0.016 | 0.012 | 0.119 | 0.226 | 0.226 | 0.015 | 0.175 | 0.275 |
| SOI total | ~ | talk ATTR | 0.617 | 0.624 | 0.097 | 0.323 | 0.882 | -0.05 | -0.05 | 0.017 | -0.11 | 0.018 |
| SOI total | ~ | talk VTL | 0 | 0 | 0 | 0 | 0 | -0.47 | -0.47 | 0.012 | -0.53 | -0.43 |
| SOI total | ~ | talk range | 0.358 | 0.357 | 0.051 | 0.2 | 0.502 | 0.103 | 0.103 | 0.011 | 0.076 | 0.144 |
| SOI total | ~ | WHR | 0.786 | 0.821 | 0.048 | 0.669 | 0.974 | 0.018 | 0.018 | 0.007 | -0.01 | 0.039 |
| SOI total | ~ | height | 0.079 | 0.085 | 0.027 | 0.025 | 0.193 | 0.192 | 0.192 | 0.017 | 0.144 | 0.245 |
| SOI total | ~ | weight | 0.281 | 0.293 | 0.074 | 0.136 | 0.728 | -0.12 | -0.12 | 0.016 | -0.16 | -0.04 |
| SOI total | ~ | age | 0.947 | 0.915 | 0.064 | 0.617 | 0.994 | 0.008 | 0.008 | 0.012 | -0.03 | 0.056 |
|  |  |  |  |  |  |  |  |  |  |  |  |  |
| JK = jackknife mean estimation, SD = jackknife estimation SD, min. = jackknife minimum, max. = jackknife maximum,~~ indicates correlation, ~ regression coefficient, p (R)= robust p values from permutation test, std. = standardized parameter value, WHR=waist-to-hip ratio, ATTR = attractiveness, VTL = apparent vocal tract length | | | | | | | | | | | | |
